# Multiomics Analyses of HNF4α Protein Domain Function during Human Pluripotent Stem Cell Differentiation

**DOI:** 10.1016/j.isci.2019.05.028

**Published:** 2019-05-24

**Authors:** Yu Wang, Michael H. Tatham, Wolfgang Schmidt-Heck, Carolyn Swann, Karamjit Singh-Dolt, Jose Meseguer-Ripolles, Baltasar Lucendo-Villarin, Tilo Kunath, Timothy R. Rudd, Andrew J.H. Smith, Jan G. Hengstler, Patricio Godoy, Ronald T. Hay, David C. Hay

**Affiliations:** 1 Medical Research Council Centre for Regenerative Medicine, University of Edinburgh, 5 Little France Drive, Edinburgh, Scotland EH16 4UU, UK; 2 Centre for Gene Regulation and Expression, School of Life Sciences, University of Dundee, Dundee DD1 5EH, UK; 3 Leibniz Institute for Natural Product Research and Infection Biology eV-Hans-Knoll Institute, Jena, Germany; 4 National Institute for Biological Standards and Control (MHRA), Blanche Lane, South Mimms, Hertfordshire EN6 3QG, UK; 5 IfADo-Leibniz Research Centre for Working Environment and Human Factors at the Technical University Dortmund, Dortmund, Germany

**Keywords:** Biological Sciences, Cell Biology, Developmental Biology, Stem Cells Research

## Abstract

During mammalian development, liver differentiation is driven by signals that converge on multiple transcription factor networks. The hepatocyte nuclear factor signaling network is known to be essential for hepatocyte specification and maintenance. In this study, we have generated deletion and point mutants of hepatocyte nuclear factor-4alpha (HNF4α) to precisely evaluate the function of protein domains during hepatocyte specification from human pluripotent stem cells. We demonstrate that nuclear HNF4α is essential for hepatic progenitor specification, and the introduction of point mutations in HNF4α′s Small Ubiquitin-like Modifier (SUMO) consensus motif leads to disrupted hepatocyte differentiation. Taking a multiomics approach, we identified key deficiencies in cell biology, which included dysfunctional metabolism, substrate adhesion, tricarboxylic acid cycle flux, microRNA transport, and mRNA processing. In summary, the combination of genome editing and multiomics analyses has provided *valuable* insight into the diverse functions of HNF4α during pluripotent stem cell entry into the hepatic lineage and during hepatocellular differentiation.

## Highlights

• Deletion of HNF4α's DNA-binding domain blocks hepatic progenitor formation
• HNF4α′s C-terminal SUMO consensus motif plays roles in hepatocyte specification
• Multiomics analyses reveal various processes regulated by HNF4α during differentiation

## Graphical Abstract

**Figure:**
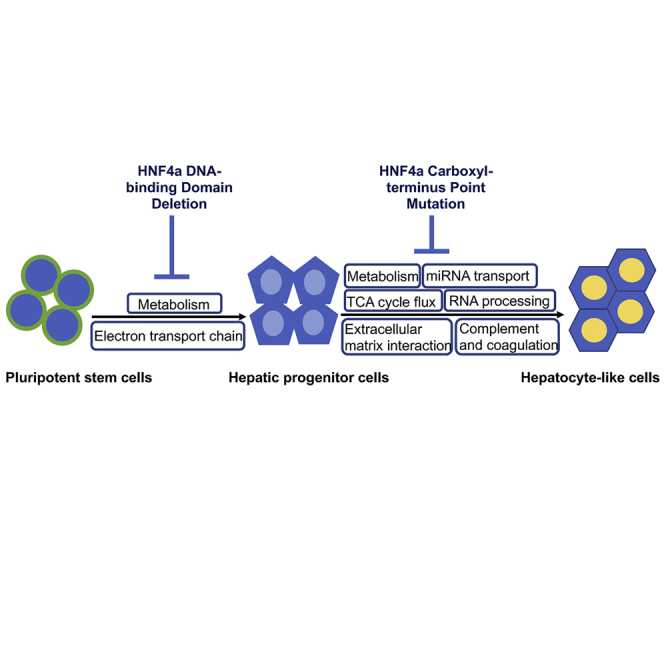

## Introduction

Directed differentiation of human pluripotent stem cells offers robust systems to study gene function during human development. Defined and efficient human hepatic differentiation systems have been developed and automated, allowing detailed mechanistic studies to be performed (Hay et al., 2007, Hay et al., 2008, Lucendo-Villarin et al., 2017, Meseguer-Ripolles et al., 2018, Rashidi et al., 2018, Si-Tayeb et al., 2010, Sullivan et al., 2010, Szkolnicka et al., 2014). These models have been sophisticated further using modern genome editing systems. Recently, pluripotent stem cells with an albumin reporter system have facilitated the discovery of key players involved in hepatocyte maturation (Li et al., 2018). In our study, the clustered regularly interspaced short palindromic repeats (CRISPR)/CRISPR-associated (Cas) endonuclease nickase was employed for hepatocyte nuclear factor-4alpha (HNF4α) genome editing in human pluripotent stem cells.

HNF4α is a highly conserved transcription factor of the nuclear receptor superfamily (Sladek et al., 1990). It is a modular protein containing five functional regions. Regions A and B contain the N-terminal transactivation domain (AD-1), region C is a highly conserved zinc finger DNA-binding domain (DBD), region D is a flexible hinge, region E is a multifunctional ligand-binding domain containing the second transactivation domain (AD-2), and the repression region is located in region F (Lau et al., 2018). Both transactivation domains are important to HNF4α-driven gene transcription (Dhe-Paganon et al., 2002). To understand which regions of the HNF4α protein are required for directing cell specification and maturation, we used CRISPR-Cas9-based genome editing technology to modify the DBD in the amino terminus, and AD-2 in the carboxy terminus.

Previous genome-wide location analysis revealed that HNF4α was bound to ∼12% of genes in human hepatocytes, which was greater than other hepatocyte nuclear factors (Odom et al., 2004). Notably, 42% of the actively transcribed genes occupied by RNA polymerase II were bound by HNF4α, demonstrating its central role in hepatocyte biology (Odom et al., 2004). More recent researches showed that HNF4α was essential to the formation of hepatic progenitors during human pluripotent stem cell differentiation (DeLaForest et al., 2011, DeLaForest et al., 2018). In these studies, HNF4α was required for the recruitment of RNA polymerase II to genes that were specifically expressed at the hepatic progenitor stage (DeLaForest et al., 2018).

Given its potent activity, HNF4α is regulated at numerous levels, which include nuclear receptor interaction, microRNA (miRNA) regulation, and post-translational modification (Lau et al., 2018, Ramamoorthy et al., 2012, Yokoyama et al., 2011). A number of post-translational modifications have been shown to regulate HNF4α protein stability, function, and subcellular localization (Jiang et al., 1997, Ktistaki et al., 1995, Viollet et al., 1997, Yokoyama et al., 2011, Zhou et al., 2012). In addition, recent studies highlighted that HNF4α protein stability is regulated post-translationally through interaction with heat shock protein 90 β interaction (Jing et al., 2017). In these studies, we were particularly interested in HNF4α′s post-translational modification by the Small Ubiquitin-like Modifier (SUMO) at a consensus motif in the AD-2 domain of the carboxy terminus (Zhou et al., 2012).

This study examined HNF4α protein domain function during hepatoblast and hepatocyte specification from pluripotent stem cells. Isogenic pluripotent stem cell lines, which possessed a truncation or point mutations in HNF4α, were created and compared with a wild-type (WT) line. Pluripotent stem cells that possessed the DNA-binding domain deletion mutant of HNF4α displayed defects in endoderm and hepatic progenitor specification, whereas HNF4α point mutants failed to form functional hepatocytes, with defects in cell metabolism, adhesion, tricarboxylic acid (TCA) cycle flux, miRNA transport, and mRNA processing detected.

## Results

### Generation and Characterization of *HNF4α*-Edited Pluripotent Stem Cell Lines

**Figure 1:**
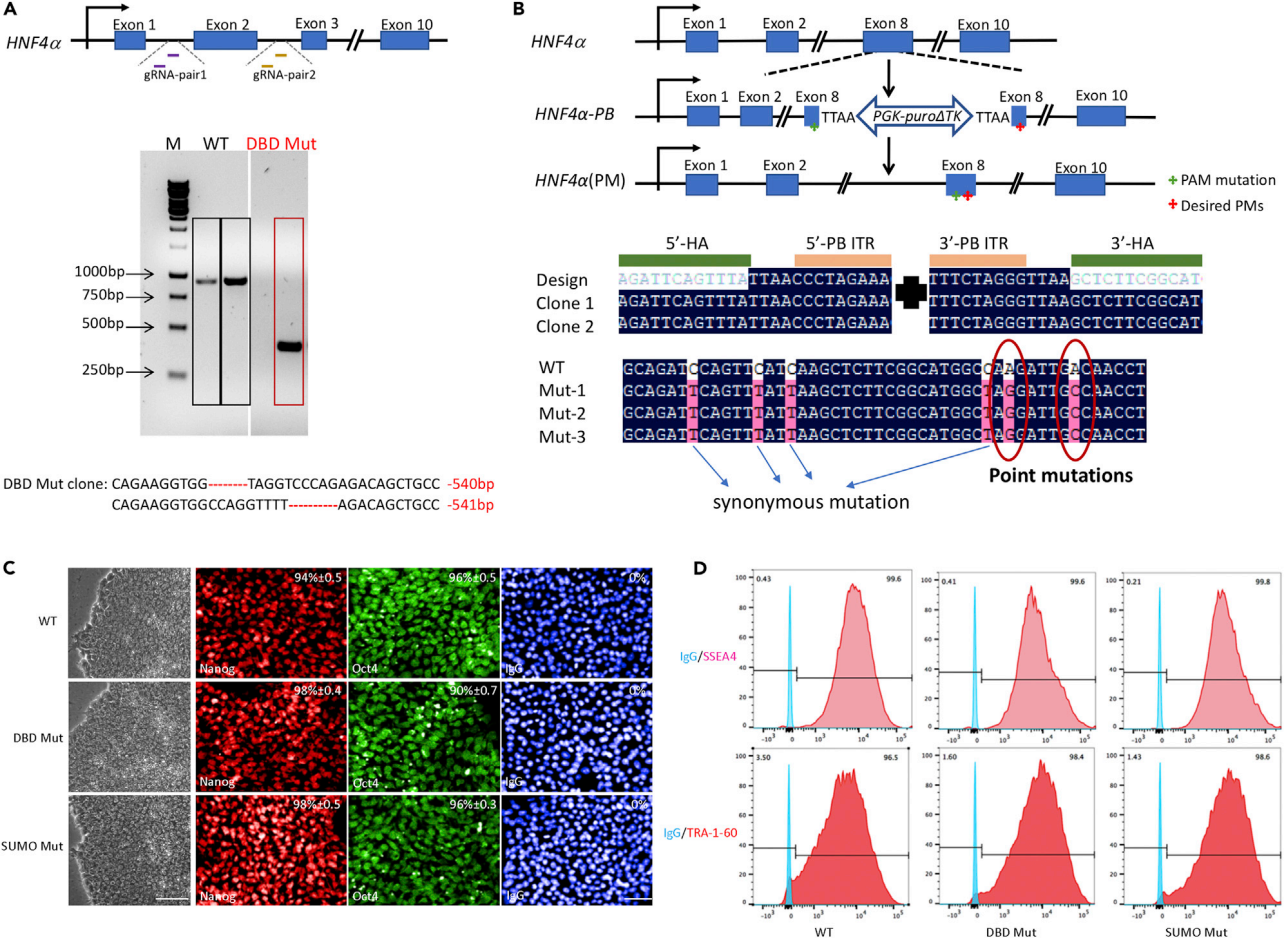
The Generation and Characterization of *HNF4α* Genome-Edited Cell Lines (A) Two guide RNA pairs targeting introns 1 and 2 were used to delete exon 2 in *HNF4α* (top panel). A homozygous deletion clone was identified by amplifying the targeted region (middle panel). Sequencing confirmed the deletion mutant (DBD Mut) clone had a 540/541-bp deletion in each allele (bottom panel). See Tables S5 and S6 for further details. (B) A piggyBac-based targeting vector was used in combination with Cas9 nickases to introduce desired point mutations into *HNF4α* (top panel). The targeted clones incorporated the selection cassette (middle panel). This selection cassette is contained within the piggyBac transposon and consists of a positive-negative selection marker (puro-tk) expressed from a constitutively active promoter (PGK). Post excision of the transposon, the locus was modified seamlessly (bottom panel). PAM, protospacer-adjacent motif; HA, homology arm; PB, piggyBac, 5′-PB ITR and 3′-PB ITR are 5′ and 3′ piggyBac inverted terminal repeats flanked by the TTAA direct repeats. See Tables S5 and S6 for further details. (C) Representative images of cellular morphology, immunofluorescences of NANOG and OCT4. One wild-type (WT) clone, one DBD Mut clone, and one point-mutated (SUMO Mut) clone was selected for characterization. IgG was used as a negative control. The percentage was calculated using four random fields of view. Scale bar, 100 μm for phase contrast and 50 μm for immunostaining images. (D) Flow cytometry of SSEA4- and TRA-1-60-expressing cells in the WT, DBD Mut, and SUMO Mut clones. IgG was used as a negative control. N = 3 independent experiments.

To reduce potential off-target effects, we used paired CRISPR-Cas9 nickases to edit *HNF4α* (Ran et al., 2013). Two pairs of Cas9 nickases were utilized to delete the consensus exon 2 in *HNF4α* (Figure 1A, top panel). The PCR product from amplification of the targeted region in a homozygous deletion mutant cell line was smaller than the WT control (Figure 1A, middle panel). There was a 540- and 541-bp deletion in each allele, respectively, which was confirmed by sequencing (Figure 1A, bottom panel). In parallel, two point mutations (K365R and D367A) were introduced into the SUMOylation consensus motif in the C terminus of HNF4α using Cas9 nickase and a piggyBac-based targeting vector (Zhou et al., 2012, Yusa, 2013). PCR genotyping followed by sequencing identified the insertion of the selection cassette in the targeted clones. The piggyBac repeats were inserted between 5′- and 3′-homology arms (Figure 1B, middle panel). Post removal of the selection cassette, sequencing results confirmed the seamless editing of this locus. In the point mutated clones, two point mutations were introduced into the *HNF4α* gene (AAG to AGG, K365R; GAC to GCC, D367A). Four synonymous mutations were also introduced to allow the integration of piggyBac (TTAA site) and to disrupt the protospacer-adjacent motif (PAM) sequence for Cas9 nickases (Figure 1B, bottom panel).

Following genome editing, one WT, one homozygous deletion mutant (DBD Mut), and one point mutated clone (SUMO Mut) were expanded, differentiated, and characterized in detail. Similar to the WT clone, the DBD Mut and SUMO Mut clones possessed typical pluripotent stem cell morphology, more than 90% cells expressed NANOG and OCT4, as well as cell surface markers SSEA4 and TRA-1-60 (Figures 1C and 1D). See Table S7 for further details.

### Hepatocyte Differentiation Was Perturbed in *HNF4α*-Edited Pluripotent Stem Cells

**Figure 2:**
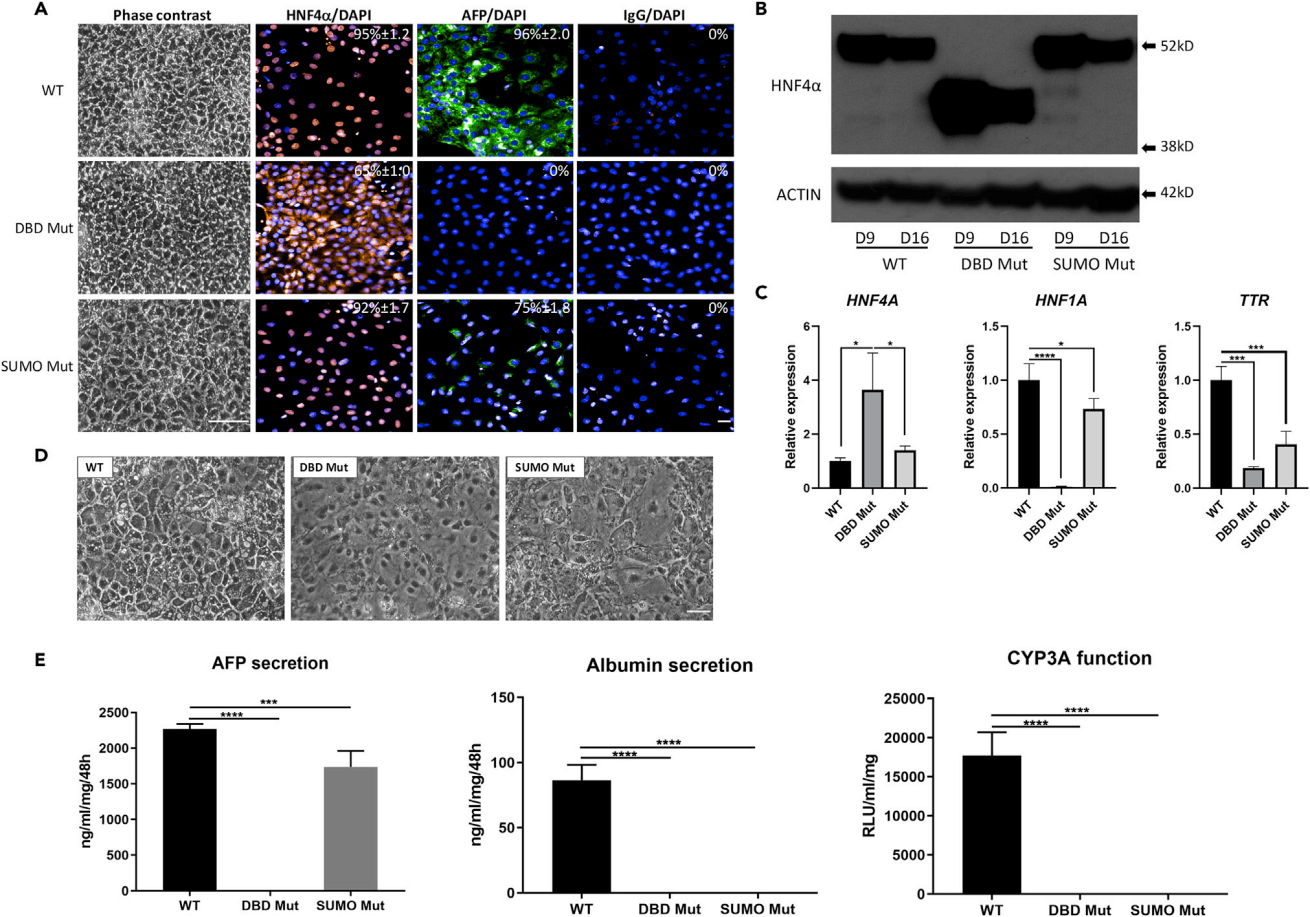
Hepatocyte Differentiation of *HNF4α* Genome-Edited Pluripotent Stem Cells (A) Morphology and immunostaining of HNF4α and AFP in the differentiated cells at hepatic progenitor stage. Scale bar, 100 μm for phase contrast and 50 μm for immunostaining images. (B) Western bloting for HNF4α in differentiated cells at days 9 (hepatic progenitor stage) and 16 (hepatocyte like cell). ACTIN was used as a loading control. See Table S7 for further details. (C) Real-time PCR quantification of HNF4α, HNF1α, and transthyretin (TTR) mRNA levels in hepatic progenitor stage (day 9) cells. Data were normalized to the housekeeping gene ACTB and expressed relative to the WT cells. The results shown represent three biological replicates, and error bars represent SD. *p < 0.05, ***p < 0.001, ****p < 0.0001; one-way ANOVA with Tukey post hoc test. See Table S8 for further details. (D) Morphology of differentiated cells at hepatocyte-like cell stage (differentiation day 18). Scale bar, 100 μm. (E) ELISA quantification of AFP and albumin secretion in differentiated cells at hepatocyte-like cell stage and quantification of cytochrome P450 3A (CYP3A) activity. Data represented three biological replicates, and error bars represented SD. ***p < 0.001, ****p < 0.0001; one-way ANOVA with Tukey post hoc test.

To study the effect of editing *HNF4α* in liver cells, we differentiated WT, DBD Mut, and SUMO Mut clones towards hepatic lineage using a stage-wise differentiation protocol (Meseguer-Ripolles et al., 2018, Wang et al., 2017). WT human embryonic stem cells transited through definitive endoderm stage (differentiation day 3) (Figures S1A and S1B) and then gave rise to polygonal hepatic progenitors (differentiation day 9) (Figure 2A). In contrast, the DBD Mut cells displayed cytoplasmic HNF4α, prolonged endoderm differentiation, and failed to commit to hepatoblast lineage, demonstrated by the lack of alpha fetoprotein (AFP) and CCAAT enhancer-binding protein alpha (CEBPA) expression (Figures 2A and S1). In contrast, the SUMO Mut cells were comparable to the WT hepatoblast in terms of morphology, demonstrating nuclear HNF4α, albeit with reduced levels of HNF1α, AFP, and CEBPA expression (Figures 2A, 2C, and S1C).

Western bloting demonstrated that the DBD Mut hepatic progenitor cell expressed a truncated form of HNF4α, which was about 10 kDa smaller than the WT or SUMO Mut HNF4α (Figure 2B). Sequencing of HNF4α cDNA in the DBD Mut cells confirmed that exons 2 and 3 were skipped in these cells, resulting in the truncated form of HNF4α (Figure S2). A decrease in HNF4α expression was observed in all cell lines between days 9 and 16, which was consistent with previous studies in WT cells (Zhou et al., 2012) (Figure 2B). In addition, real-time PCR confirmed higher levels of HNF4α mRNA in the DBD Mut progenitor cells than in the WT or SUMO Mut cells (Figure 2C). Despite this, HNF4α in the DBD Mut cells failed to transactivate HNF1α and AFP gene expression and yielded lower levels of transthyretin (*TTR*) (Figures 2A and 2C).

In addition, neither the DBD Mut nor the SUMO Mut cells could produce hepatocyte-like cells. Their morphologies were different from each other and the WT control cells (Figure 2D). Functionally, the DBD Mut cells had no detectable albumin or AFP secretion, or basal cytochrome P450 (CYP) 3A activity (Figure 2E), demonstrating failed hepatoblast and hepatocyte differentiation. The SUMO Mut cells secreted AFP at lower levels than the WT cells; however, they did not exhibit albumin secretion or basal CYP3A activity when hepatocyte specification was induced, demonstrating failed hepatocyte specification (Figure 2E).

### Disruption of Cellular Bioenergetics in *HNF4α*-Edited Cells during Cell Specification

**Figure 3:**
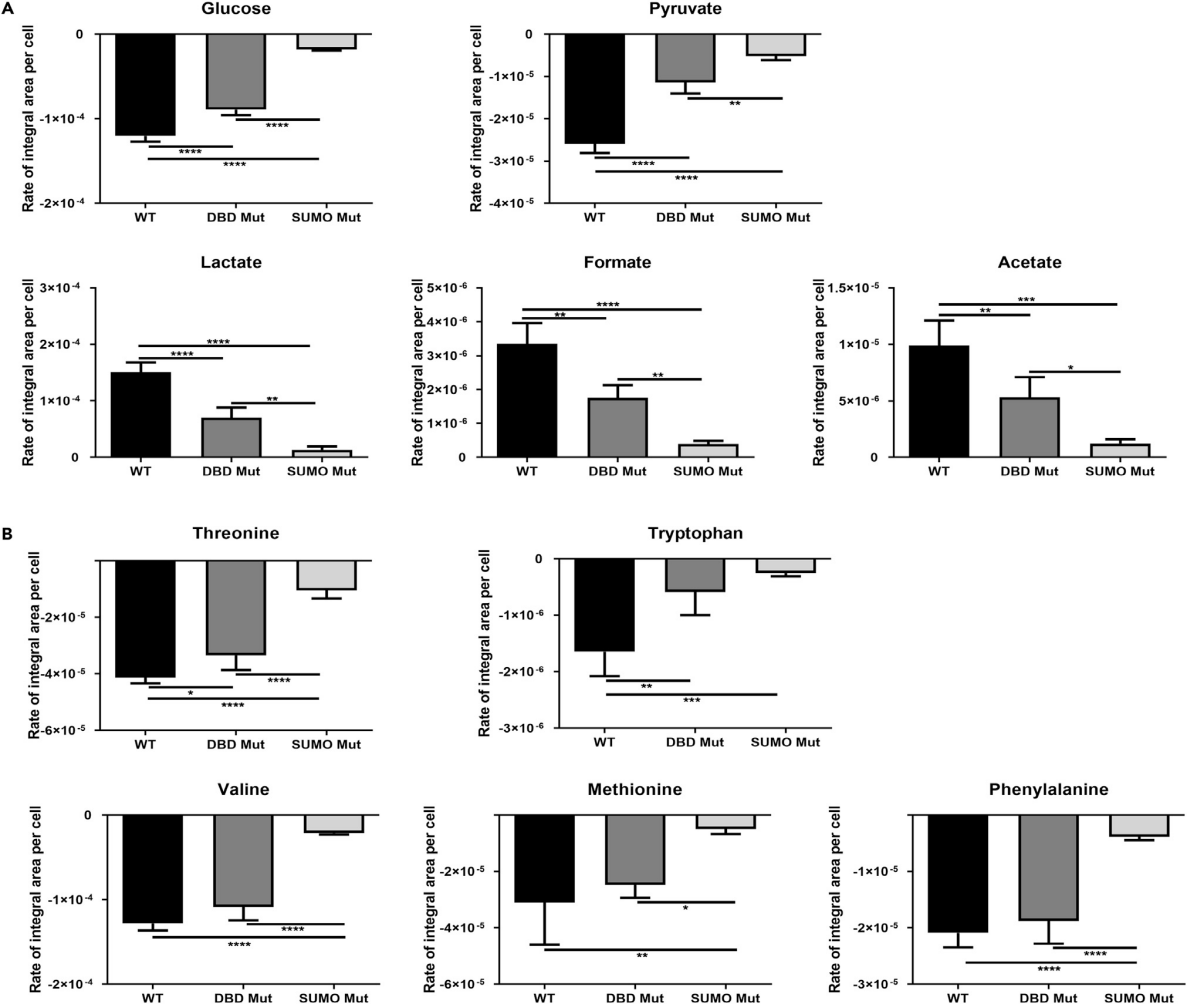
Metabolomic Studies in Hepatic Progenitor Stage Cells from WT and *HNF4α* Genome-Edited Pluripotent Stem Cells (A) Metabolic rates of glucose, pyruvate, lactate, formate, and acetate in the WT, DBD Mut, and SUMO Mut cells. (B) Metabolic rates of essential amino acids threonine, tryptophan, valine, methionine, and phenylalanine in the WT, DBD Mut, and SUMO Mut cells. Data represent three biological replicates, and error bars represent SD. *p < 0.05, **p < 0.01, ***p < 0.001, ****p < 0.0001; one-way ANOVA with Tukey post hoc test.

To gain an understanding of the DBD Mut and SUMO Mut cells at the hepatic progenitor stage (day 9), we performed non-targeted profiling of metabolites using ^1^H nuclear magnetic resonance metabolomics (Patti et al., 2012). The metabolic rate for each metabolite was quantified by normalizing the signal intensity against the cell number at the detected time point. The unit for the metabolic rate was then recorded as rate of integral area per cell. Negative and positive rate values indicate consumption and production respectively. The DBD Mut and SUMO Mut cells had a significantly lower consumption rate for both glucose and pyruvate than the WT control (Figure 3A). This was confirmed by reduced production rate of lactate, formate, and acetate (Figure 3A) and suggests compromised glycolysis and pyruvate oxidation or cycling in both cell lines. In addition, the DBD Mut and SUMO Mut cells possessed a lower consumption rate of the essential amino acids threonine and tryptophan compared with the WT cells (Figure 3B). The SUMO Mut cells also demonstrated slower consumption of valine, methionine, and phenylalanine than both WT and DBD Mut cells (Figure 3B). Notably, the DBD Mut cells had the highest consumption rate of isoleucine among the three cell types (Figure S3). The WT cells had significantly increased production of alanine and consumption of tyrosine and t-methylhistidine than the other cell lines (Figure S3). These data demonstrated that amino acid metabolism and bioenergetics were disrupted in HNF4α DBD Mut and SUMO Mut cells.

### Transcriptomic Analysis of WT and *HNF4α*-Edited Hepatic Progenitor Stage Cells

**Figure 4:**
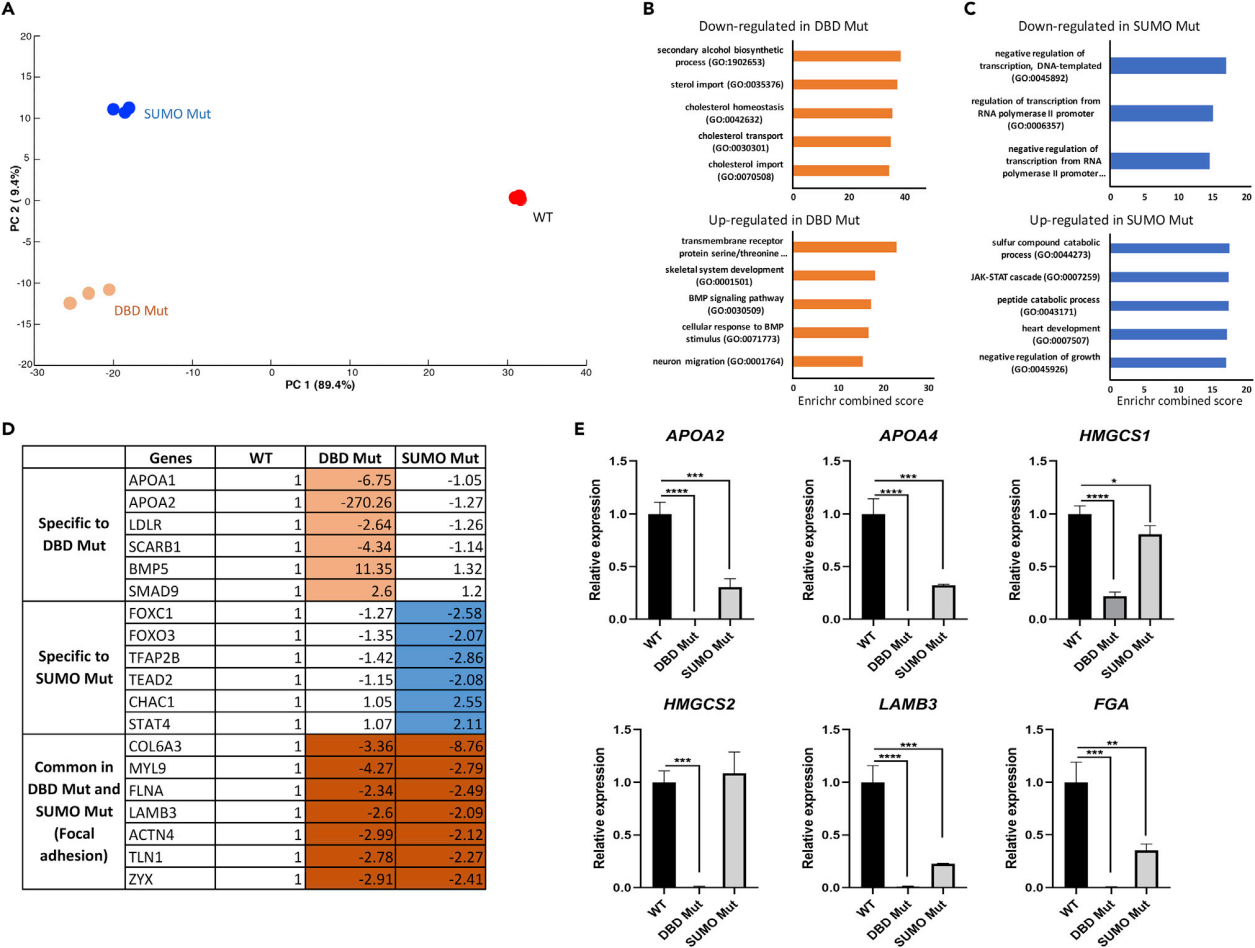
Transcriptomic Analysis of Hepatic Progenitor Stage Cells from WT and *HNF4α* Genome-Edited Pluripotent Stem Cells (A) Principal-component analysis of the three types of cells (n = 3 replicates) based on 1,000 genes with the highest variance. (B) Top five enriched biological processes of the significantly down- or up-regulated genes specific in the DBD Mut cells. Pathway enrichment was analyzed using Enrichr web server. (C) Top five enriched biological processes of the significantly down- or up-regulated genes unique to the SUMO Mut cells. Pathway enrichment was analyzed using Enrichr web server. (D) Fold changes of representative genes in the DBD Mut and SUMO Mut cells compared with the WT control from the microarray analysis. The gene expression levels have been log2 transformed. (E) Real-time PCR results of representative genes *APOA2*, *APOA4*, *HMGCS1*, *HMGCS2*, laminin subunit beta 3 (*LAMB3*), and fibrinogen alpha chain (*FGA*). Data were normalized to the housekeeping gene ACTB and expressed relative to the WT cells. The results shown represent three biological replicates, and error bars represent SD. *p < 0.05, **p < 0.01, ***p < 0.001, ****p < 0.0001; one-way ANOVA with Tukey post hoc test. See Table S8 for further details.

The cellular phenotypes we observed highlighted that proper HNF4α function was vital to cell metabolism and hepatocyte specification. To further understand the effect of *HNF4α* editing on hepatic progenitor cell biology, transcriptomic profiles were generated for WT, DBD Mut, and SUMO Mut cells at differentiation day 9. Principal-component analysis (PCA) of 1,000 genes with the highest variance demonstrated a clear difference among DBD Mut, SUMO Mut, and WT (Figures 4A and S4). A set of 1,310 differentially expressed genes (DEGs) was defined for further evaluation, which were either differential between the DBD Mut and WT cells or between the SUMO Mut and WT cells (p value <0.05; 2-fold threshold, false discovery rate adjusted; Table S1). Gene ontology (GO) enrichment analysis using the Enrichr web server (Chen et al., 2013, Kuleshov et al., 2016) showed that the 452 down-regulated genes unique to DBD Mut cells were enriched in biological processes such as “sterol import (GO:0035376)” and “cholesterol homeostasis (GO: 0042632)” (Figure 4B and Table S2). Representative genes included apolipoprotein A1 (*APOA1*), *APOA2,* and low-density lipoprotein receptor (*LDLR*) (Figure 4D and Table S2). The expression of a number of significantly down-regulated genes was confirmed by real-time PCR results, including *APOA2*, *APOA4*, and 3-hydroxy-3-methylglutaryl-CoA synthase 1 and 2 (*HMGCS1* and *HMGCS2*) (Figure 4E). We also detected 215 unique up-regulated genes in the DBD Mut cells. Those were most enriched in pathways like “transmembrane receptor protein serine/threonine kinase signaling pathway (GO:0007178)” and “skeletal system development” (Figure 4B and Table S2). The representative genes included bone morphogenetic protein 5 (*BMP5*) and *SMAD9* (Figure 4D and Table S1). Interestingly, all up-regulated genes in the DBD Mut cells compared with the WT control showed features for ectoderm differentiation (Table S2). When combined, these specific DEGs in the Del Mut cells were most enriched in cholesterol homeostasis, extracellular matrix (ECM) organization, alpha-amino acid catabolic process, and cytoskeleton organization regulation (Table S2). Taken together, the HNF4α DBD domain is essential for the expression of genes important for cell metabolism, ECM, and cytoskeleton organization during hepatoblast specification.

We also analyzed the specific DEGs in the SUMO Mut cells to understand the effect of modifying the HNF4α SUMO motif. We detected 260 down-regulated genes specific to the SUMO Mut cells. Those were enriched for biological processes including “negative regulation of transcription (GO:0045892)” and “regulation of transcription from RNA polymerase II promoter (GO:0006357)” (Figure 4C and Table S2). Genes such as the forkhead box protein C1 and O3 (*FOXC1* and *FOXO3*) were significantly down-regulated (Figure 4D and Table S1). We also detected 141 up-regulated genes enriched for processes like “sulfur compound catabolic process (GO:0044273)” and “JAK-STAT cascade (GO:0007259)” (Figure 4C and Table S2). Representative genes included ChaC glutathione-specific gamma-glutamylcyclotransferase 1 (*CHAC1*) and signal transducer and activator of transcription 4 (*STAT4*) (Figure 4D and Table S1). Combining the significantly down- and up-regulated genes together, the most enriched biological process was negative regulation of gene transcription (Table S2). Collectively, the introduced point mutations in the HNF4α SUMO consensus motif most likely affected gene transcription necessary for successful hepatocyte specification.

We also studied transcriptomic differences between the different mutant forms of HNF4α; 584 genes were down-regulated in the DBD Mut cells when compared with the SUMO Mut. They were mainly enriched in cholesterol homeostasis and lipoprotein remodeling. There were also 242 genes up-regulated in the DBD Mut cells when compared with the SUMO Mut cells. These genes demonstrated a shift towards muscle cell differentiation (Tables S1 and S2).

Further analysis revealed that 169 DEGs were down-regulated in both DBD Mut and SUMO Mut cells when compared with the WT. By referring to the Kyoto Encyclopedia of Genes and Genomes (KEGG) pathway database, the focal adhesion pathway was the only one significantly (p value < 0.05) enriched (Table S2). Representative genes encoded proteins for the basement membrane (laminin subunit beta 3), actin binding (filamin A and actinin alpha 4), and ECM binding (collagen type VI alpha 3 chain and talin 1) (Figures 4D and 4E and Table S1). There were also 74 DEGs up-regulated in both the DBD Mut and SUMO Mut cells; however, no pathway was significantly enriched. Therefore focal adhesion alterations in both DBD Mut and SUMO Mut cells probably contributed to hepatoblast and hepatocyte formation.

### Proteomic Analysis of WT and *HNF4α*-Edited Hepatic Progenitor Stage Cells

**Figure 5:**
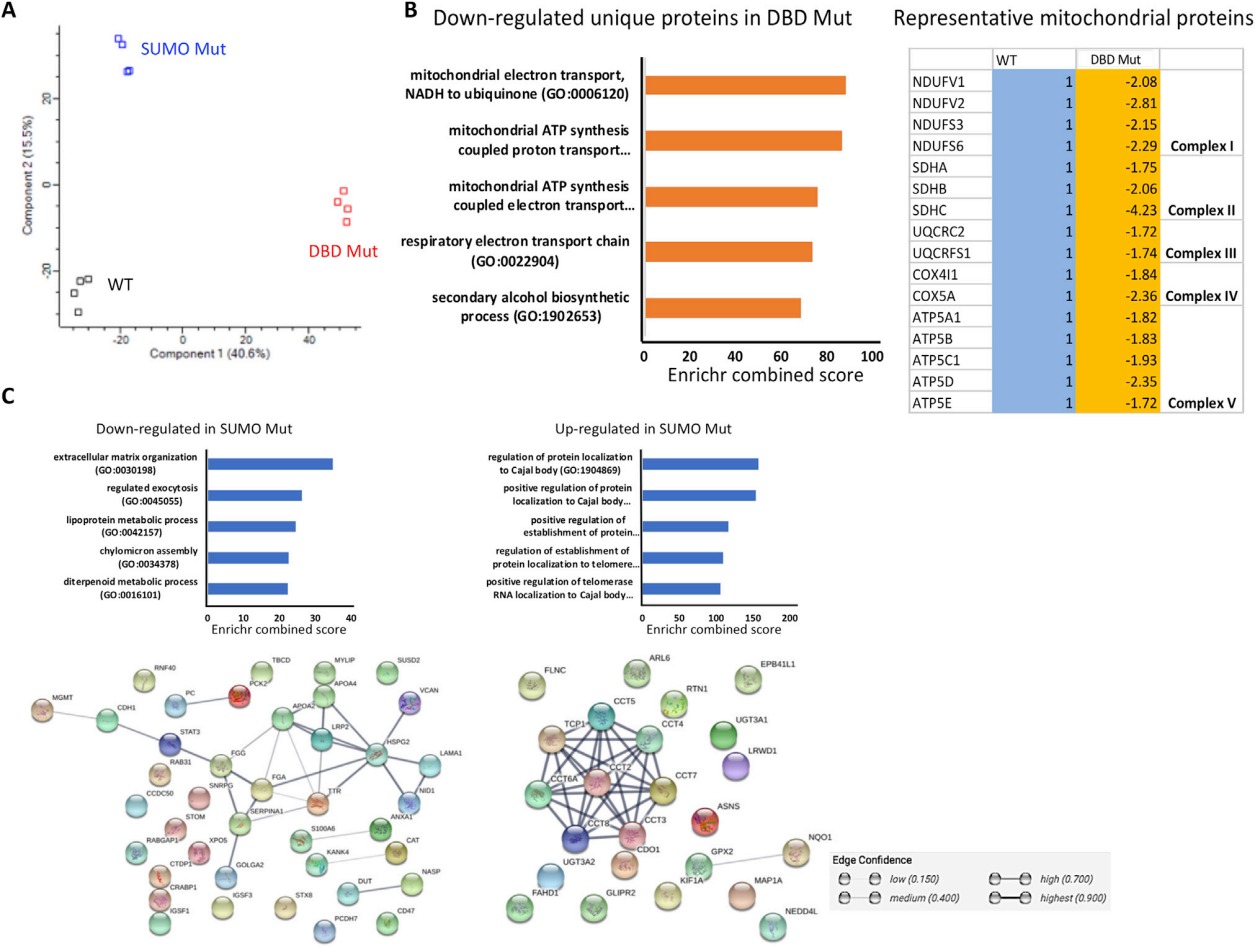
Proteomic Analysis of Hepatic Progenitor Stage Cells from WT and *HNF4α* Genome-Edited Pluripotent Stem Cells (A) Principal component analysis of the WT, DBD Mut, and SUMO Mut cells at hepatic progenitor stage (n = 4 replicates). (B) Top five enriched biological processes of the significantly down-regulated proteins specifically in the DBD Mut cells and the representative down-regulated mitochondrial proteins in the DBD Mut cells. Pathway enrichment was analyzed using Enrichr server. (C) Top five enriched biological processes and the protein-protein interaction networks of the significantly down- or up-regulated proteins in the SUMO Mut cells compared with the WT. Pathway enrichment was analyzed using Enrichr server. The protein-protein interaction networks were generated on STRING server. Network nodes represent proteins, and edges represent protein-protein associations. The bolder the edge, the higher the confidence.

Following on from the metabolic and transcriptomic studies, we examined alterations in the proteome at differentiation day 9 from WT, DBD Mut, and SUMO Mut lines. Tryptic peptides were analyzed by LC-MS/MS, and the raw MS output data was processed using the MaxQuant platform and then analyzed in Perseus software (Tyanova et al., 2015, Tyanova et al., 2016). A total of 3,639 protein groups were evaluated across all cell types. PCA analysis showed that the DBD Mut cells were distinct from the WT and SUMO Mut cells (Figure 5A and S5A). Proteins with reduced expression unique to the DBD Mut cells (Table S3), compared with WT, were involved in metabolic processes and oxidative phosphorylation (Figure 5B and Table S4). Notably, 119 of the 486 less abundant proteins specifically in the DBD Mut cells were mitochondrial proteins (Table S3). A number of subunits of Complexes I, II, III, IV, and V from the respiratory electron transport chain were down-regulated in the DBD Mut cells (Figure 5B). These included the core subunits of Complex I NADH dehydrogenase [ubiquinone] flavoprotein 1 and 2 (NDUFV1 and NDUFV2) and NADH dehydrogenase [ubiquinone] iron-sulfur protein 3 (NDUFS3) (Figure 5B and Table S3). In addition, the most down-regulated proteins in the DBD Mut included mitochondrial HMGCS2 and glycine dehydrogenase (GLDC) (Table S3). HMGCS2 catalyzes the first step of ketogenesis, whereas GLDC is involved in glycine degradation. The decreased expression of HMGCS2 and GLDC was consistent at the mRNA level, together with other metabolic enzymes such as glutamate dehydrogenase 1 (GLUD1) and aldehyde dehydrogenase 5 family member A1 (ALDH5A1) (Figures 4E and S5B). Taken together, the HNF4α DBD domain is essential to mitochondrial function and cellular metabolism.

Proteins detected at reduced levels in the SUMO Mut cells were significantly enriched for ECM organization and lipoprotein metabolic pathways (Figure 5C and Table S4). A dense network of associations was found around heparan sulfate proteoglycan 2 (HSPG2) in STRING, which is a web resource of known and predicted protein-protein interactions (Szklarczyk et al., 2015). HSPG2 encodes the perlecan protein, a core component of basement membranes. HSPG2 formed strong associations with the thyroid hormone transporter TTR, apolipoproteins APOA2 and APOA4, and the endocytic receptor low-density lipoprotein receptor-related protein 2 (LRP2), the basement membrane protein nidogen-1(NID1), ECM proteins laminin subunit alpha-1 (LAMA1) and versican (VCAN), and the alpha chain of the coagulation factor fibrinogen (FGA) (Figure 5C). Proteins that were more abundant in the SUMO Mut cells were mostly involved in the regulation of protein localization to Cajal body and telomeres (Figure 5C and Table S4). A dense network of associations was also formed by chaperonin-containing T-complex subunits (CCT1-8) (Figure 5C). Of note, SUMO Mut cells also contained differentially expressed proteins that were significantly altered for by at least 8-fold (Table S3). These proteins were involved in diverse cellular processes, including gluconeogenesis and TCA cycle flux (phosphoenolpyruvate carboxykinase 2 [PCK2]), as well as microRNA transport (Exportin 5). This suggested that the introduced point mutations in HNF4α not only affected ECM organization and cellular metabolism but also led to defects in miRNA transport.

There were 27 commonly down-regulated proteins in the DBD Mut and SUMO Mut cells when compared with the WT control. These proteins were most enriched in KEGG pathways such as complement and coagulation cascades, ECM-receptor interaction, pyruvate metabolism, and TCA cycle (Table S4). The representative proteins included pyruvate carboxylase (PC) and PCK2 (Figure S5C), which are involved in gluconeogenesis, pyruvate metabolism, and TCA cycle flux, supporting our metabolomic analysis.

### Integrating Transcriptomics and Proteomics Datasets

**Figure 6:**
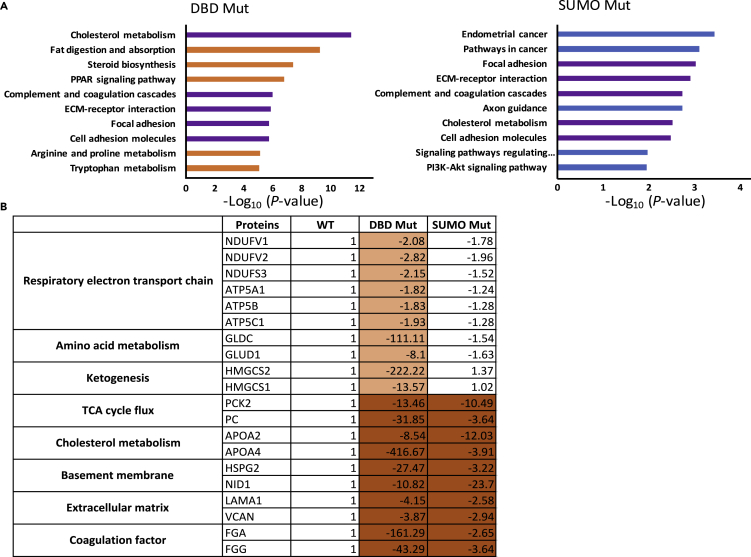
Integrated Transcriptomic and Proteomic Analysis of Hepatic Progenitor Stage Cells Differentiated from WT and *HNF4α* Genome-Edited Pluripotent Stem Cells (A) Top 10 enriched KEGG pathways in the DBD Mut cells or in the SUMO Mut cells from the overlayered analysis of both transcriptomic and proteomic datasets. (B) Representative proteins and their fold changes in expression in the DBD Mut and SUMO Mut cells when compared with WT cells. The label-free quantification intensities have been log10 transformed.

To integrate information from both transcriptomics and proteomics experiments, we re-analyzed both datasets for the edited cells (DBD Mut/WT and SUMO Mut/WT) using a free web-based multiomics data visualization application named PaintOmics 3 (http://bioinfo.cipf.es/paintomics/) (Hernandez-de-Diego et al., 2018). In the DBD Mut cells, the most enriched KEGG pathway was cholesterol metabolism, followed by pathways in peroxisome proliferator-activated receptor signaling, ECM-receptor interaction, focal adhesion, and amino acid metabolism. Five of the top 10 enriched pathways in the DBD Mut cells were also in the top 10 significantly enriched pathways in the SUMO Mut cells. They were cholesterol metabolism, complement and coagulation cascades, ECM-receptor interaction, focal adhesion, and cell adhesion molecules (Figure 6A). Based on these analyses, we summed up the representative proteins that were significantly altered in the DBD Mut cells and in both DBD Mut and SUMO Mut cells (Figure 6B and Table S3). Taken together, the DBD Mut and SUMO Mut cells shared similar dysregulated genes and proteins that function in cellular metabolism and cell-ECM interaction. Meanwhile, the DBD Mut cells had disrupted respiratory electron transport chain and metabolism, highlighting the importance of the HNF4α DBD domain.

## Discussion

The hepatocyte nuclear factor network is vital for mammalian liver development and organ homeostasis (Odom et al., 2006, Watt et al., 2003). This network controls the expression of a large number of hepatic genes, which perform a broad range of functions (Battle et al., 2006, Bolotin et al., 2010, Odom et al., 2004). In the present study, we investigated HNF4α function during hepatic specification from human pluripotent stem cells. Without its DNA-binding domain, the truncated form of HNF4α failed to make hepatic progenitor cells, and displayed disruption in key processes such as the respiratory electron transport chain and cellular metabolism. Conversely, the introduction of point mutations, in the C-terminal SUMO consensus motif of HNF4α permitted hepatic progenitor commitment, but led to failed hepatocyte specification. Key genes involved in metabolism, TCA cycle flux, miRNA transport, cell-ECM interactions, mRNA processing, and coagulation cascades were implicated in this study.

Pluripotent stem cells rely heavily on aerobic glycolysis during self-renewal, but they switch quickly to oxidative phosphorylation (OXPHOS) during mesoderm and endoderm differentiation to meet the energy demand. Interestingly, during hepatic differentiation from human pluripotent stem cells, OXPHOS has been reported to decrease when cells reach the hepatic progenitor stage, followed by an increase during hepatocyte maturation (Hopkinson et al., 2017, Jing et al., 2018). In our study, we integrated omics analyses at the hepatic progenitor stage and found that ketogenesis, amino acid metabolism, TCA flux, and cholesterol metabolism pathways were largely down-regulated in mutated cells. We propose that these alterations were contributory factors that led to failed hepatocyte specification.

Previous studies in rodents have also shown that HNF4α-null embryonic livers display defects in glycogen synthesis (Parviz et al., 2003). In addition, glucose responsiveness was abolished in murine embryoid-body-derived visceral endoderm when HNF4α was deleted (Stoffel and Duncan, 1997). In this study, we demonstrate that HNF4α is critical to glucose utilization at the hepatic progenitor stage (Figure 3A). When HNF4α was truncated or point mutated, glucose and pyruvate utilization were compromised (Figures 3A and S3). Similarly, PC and PCK2, key enzymes in pyruvate cycling, were significantly down-regulated in genome-edited cells (Figure 6B). Notably, the SUMO Mut cells showed more pronounced metabolomic changes than the DBD Mut cells (Figure 3). The reason behind this is unknown, but it could be that the knockin mutations affected HNF4α′s second transactivation domain (AD-2) leading to alterations in gene transcription.

Although the liver is regarded as one of the body's metabolic centers, less is known about the amino acid metabolism dynamics during early hepatogenesis. A recent study reported that l-valine is essential to murine liver bud growth and promotes the propagation of human hepatic progenitor organoids (Koike et al., 2017). This study did not investigate whether valine was required for human liver bud formation, but we note that valine consumption was significantly reduced in both DBD Mut and SUMO Mut cells at the hepatic progenitor stage. Although valine was not essential for hepatoblast specification in SUMO Mut cells, it is possible that valine was required for the specification of bipotent hepatic progenitors. However, further research is required to test this hypothesis.

Cell-ECM interaction also play a critical role in cellular differentiation. In the DBD Mut and SUMO Mut hepatic progenitor stage cells, we identified commonly down-regulated genes. They were enriched for focal adhesion and ECM organization pathways (Figures 4 and 6, Supplemental Information, Tables S2 and S4). Specific to the SUMO Mut cells, exportin-5 (XPO5) expression was reduced, potentially affecting precursor miRNA transport from the nucleus to the cytoplasm. We also observed down-regulation of genes that regulate gene transcription and mRNA processing, such as *FOXC1*, *FOXO3* (Figure 4D), and small nuclear ribonucleoprotein G (SNRPG) (Supplemental Information, Table S3). We believe that these data point to deficiencies in multiple cell biological processes that are instrumental in the hepatoblast to hepatocyte transition.

In summary, this study highlights the important role played by HNF4α during hepatic endoderm differentiation. System biology analyses revealed numerous potential regulatory functions for HNF4α. Those include the control of the respiratory electron transport chain, cell metabolism, pyruvate cycling, TCA cycle flux, miRNA transport, mRNA processing, and cell-ECM interaction.

### Limitations of the Study

This study was based on an *in vitro*-directed differentiation system and was not tested *in vivo*.

## Methods

All methods can be found in the accompanying Transparent Methods supplemental file.

## Supplemental Information

Document S1. Transparent Methods, Figures S1–S5, and Tables S5–S8

Table S1. DEGs, Related to Figure 4

Table S2. Gene Ontology Biological Processes, Related to Figure 4

Table S3. Proteomics, Related to Figure 5

Table S4. Gene Ontology Biological Processes for Proteomics, Related to Figure 5
