# Supplementary material for: Multiomics Analyses of HNF4α Protein Domain Function during Human Pluripotent Stem Cell Differentiation
Source: iScience. 2019 May 24;16:206–17. doi: 10.1016/j.isci.2019.05.028 (PMC6556878; doi:10.1016/j.isci.2019.05.028)
Supplement: Document S1. Transparent Methods, Figures S1–S5, and Tables S5–S8 [file mmc1.pdf]

ISCI, Volume *16*

Supplemental Information

Multiomics Analyses of HNF4α Protein Domain Function during Human Pluripotent Stem Cell Differentiation

Yu Wang, Michael H. Tatham, Wolfgang Schmidt-Heck, Carolyn Swann, Karamjit Singh-Dolt, Jose Meseguer-Ripolles, Baltasar Lucendo-Villarin, Tilo Kunath, Timothy R. Rudd, Andrew J.H. Smith, Jan G. Hengstler, Patricio Godoy, Ronald T. Hay, and David C. Hay

SUPPLEMENTAL INFORMATION

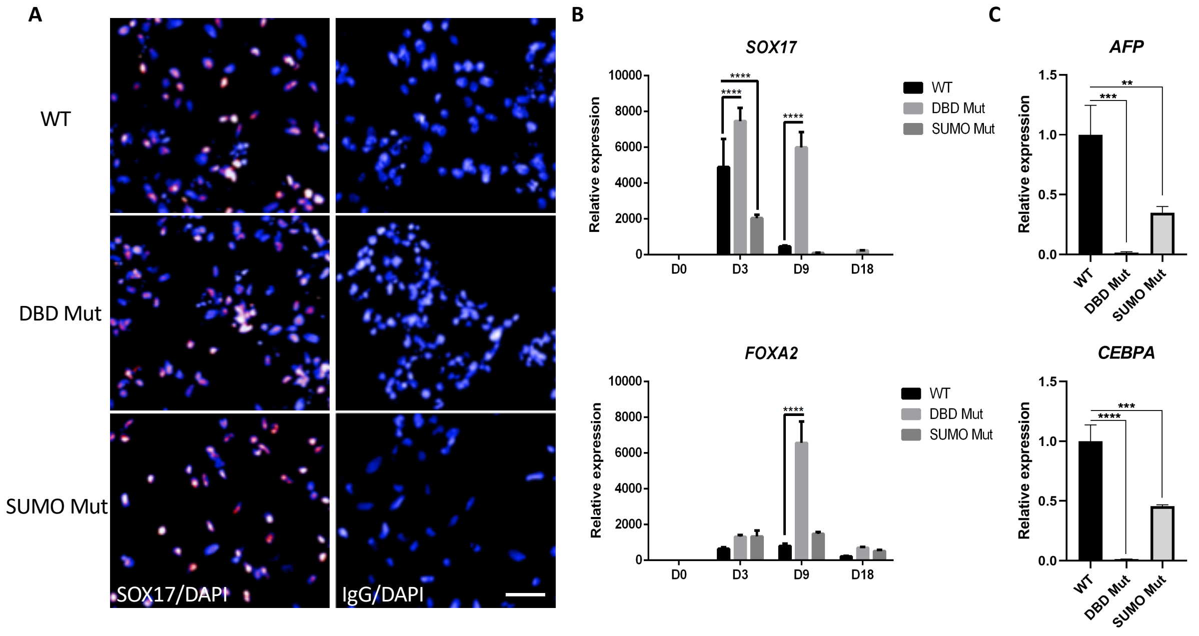

Supplemental Figure 1

Figure S1. Related to Figure 2, Differentiation towards the definitive endoderm was not affected in SUMO Mut cells, but was in DBD Mut cells.

(A) Immunostaining of SOX17 in WT, DBD Mut and SUMO Mut pluripotent stem cells differentiated toward definitive endoderm. Scale bar = 50 um.

(B) The expression levels of *SOX17* and *FOXA2* during hepatic differentiation. Data were normalized to the housekeeping gene ACTB and expressed relative to day 0 cells. The results shown represent three biological replicates and error bars represent SD. **p < 0.01, *** p < 0.001, **** p < 0.0001; two-way ANOVA with Tukey post hoc test.

(C) Real-time PCR quantification of AFP and CEBPA mRNA level. Data were normalized to the housekeeping gene ACTB and expressed relative to the WT cells. The results shown represent three biological replicates and error bars represent SD. **p < 0.01, *** p < 0.001, **** p < 0.0001; one-way ANOVA with Tukey post hoc test.

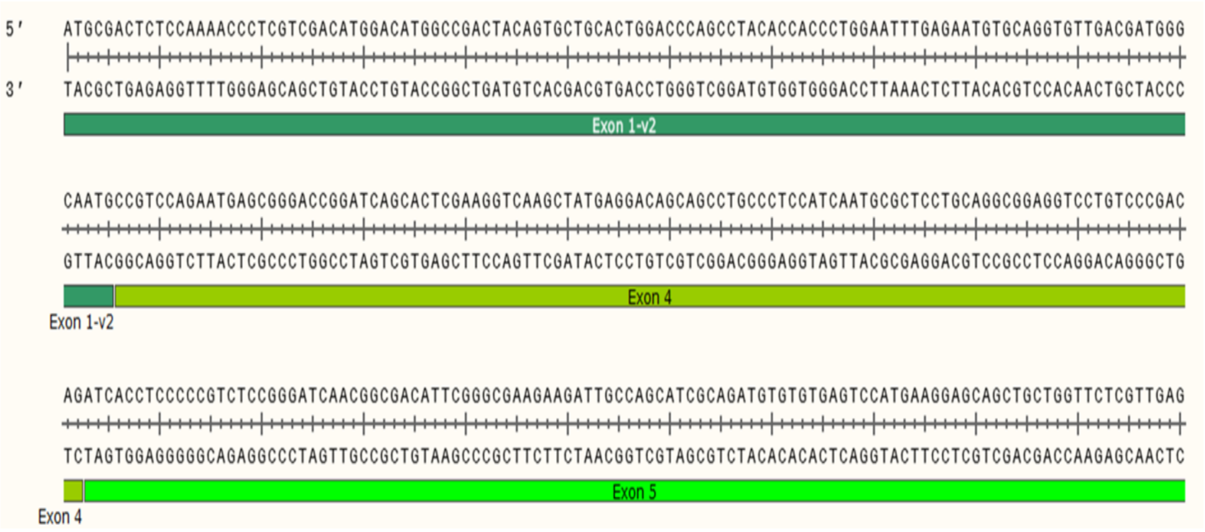

Supplemental Figure 2

Figure S2. Related to Figure 2B, Exons 2 and 3 were skipped in HNF4α DBD Mut cells.

Sequencing of HNF4α cDNA in the DBD Mut cells at hepatic progenitor stage showed exon 2 and 3 were skipped.

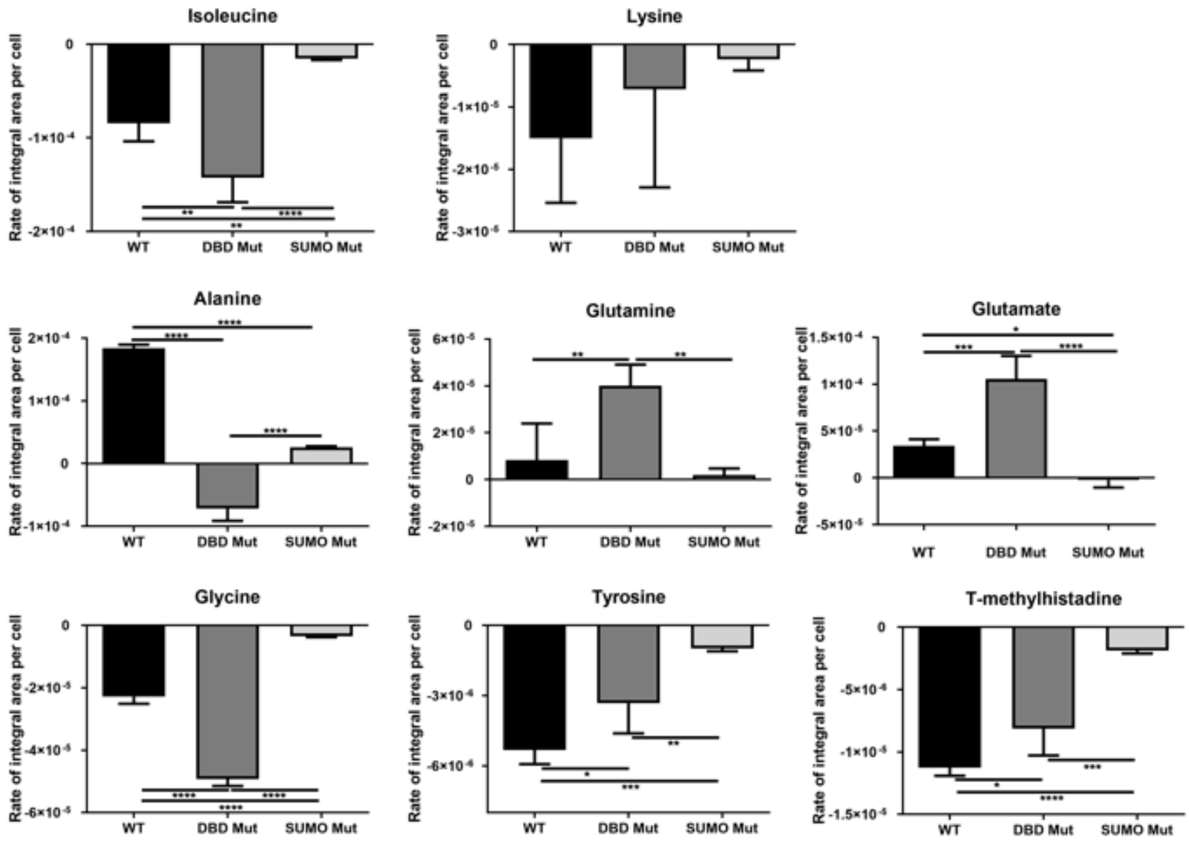

Supplemental Figure 3

Figure S3. Related to Figure 3, Amino acid metabolism was disrupted in the DBD Mut and SUMO Mut hepatoblasts.

Consumption and production rates of essential amino acids (isoleucine and lysine) and non-essential amino acids (alanine, glutamine, glutamate, glycine, tyrosine and t-methylhistadine) in WT, DBD Mut

and SUMO Mut cells at day 9 in differentiation. Data represent three biological replicates and error bars represent SD. * p < 0.05, ** p < 0.01, *** p < 0.001, **** p < 0.0001; one-way ANOVA with Tukey post hoc test.

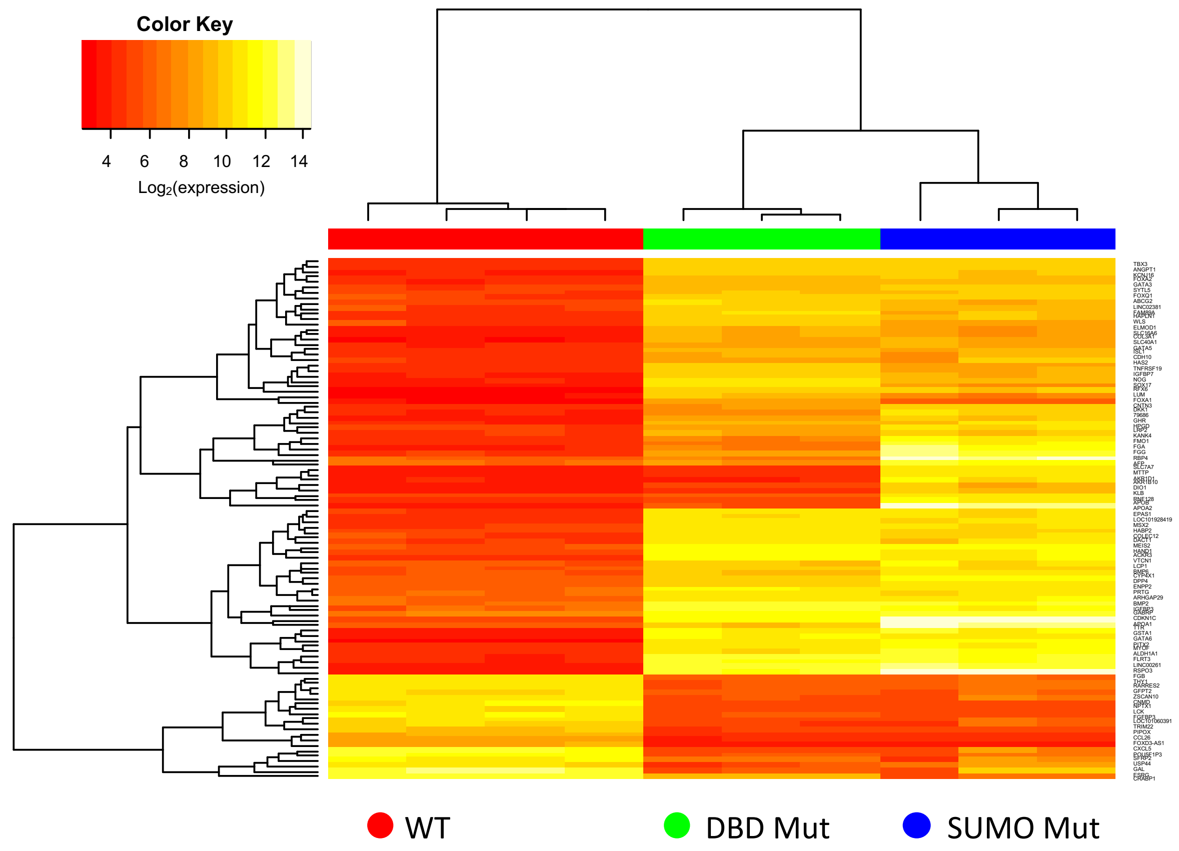

Supplemental Figure 4

Figure S4. Related to Figure 4, Heat map representation of the 100 genes with highest variance in WT, DBD Mut and SUMO Mut cells at the hepatic progenitor stage.

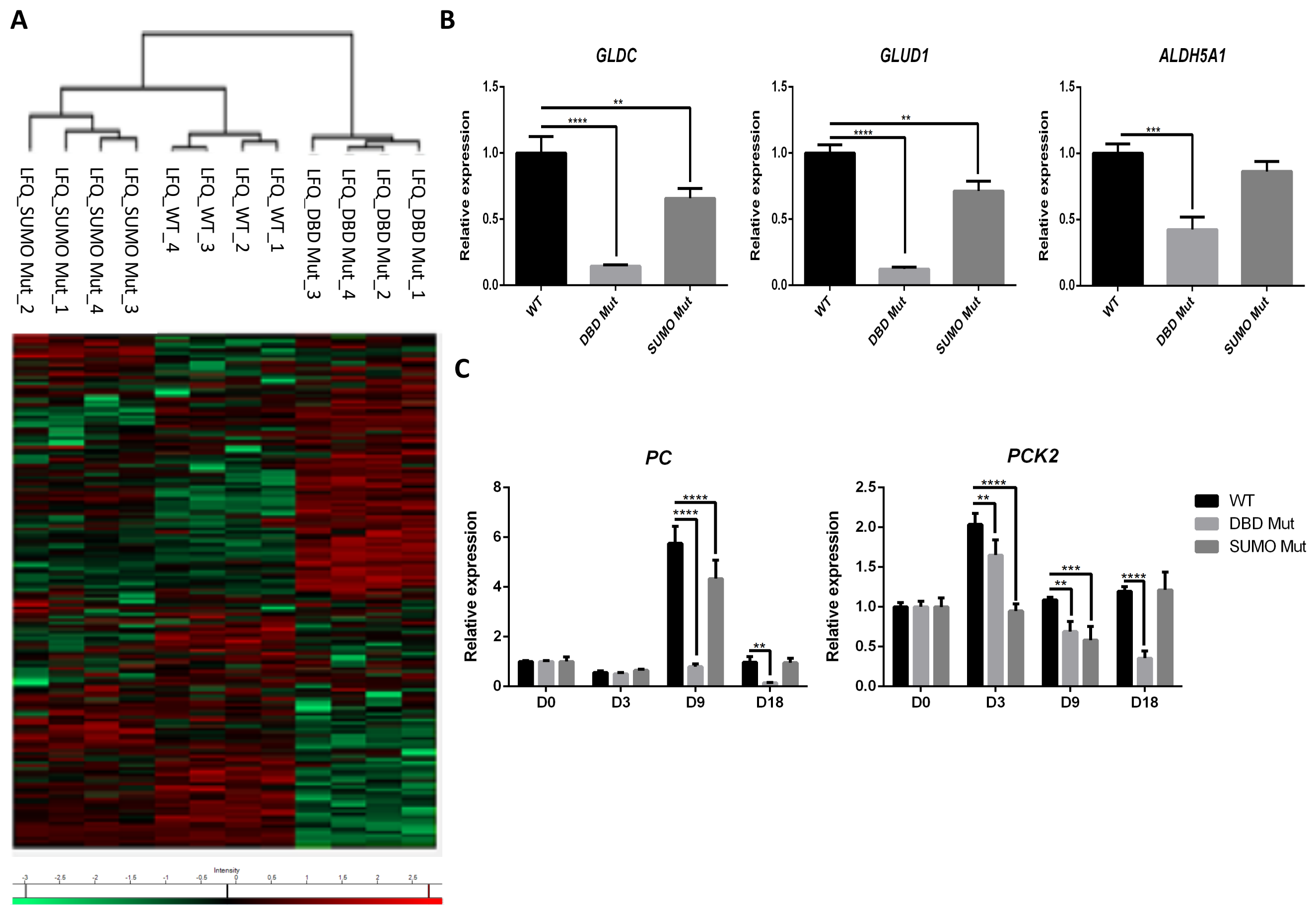

Supplemental Figure 5

Figure S5. Related to Figure 5, Heat map representation of the proteomics dataset and the expression of representative metabolic enzymes.

(A) Heat map of proteomics analysis of the WT, DBD Mut and SUMO Mut cells at the hepatic progenitor stage. The LFQ intensities were log10 transformed, then z-scored and normalized by row. (B) The expression of metabolic enzymes GLDC, GLUD1 and ALDH5A1 at mRNA level in the progenitor stage cells. Data were normalized to the housekeeping gene ACTB and expressed relative to the WT cells. The results shown represent three biological replicates and error bars represent SD. **p < 0.01, *** p < 0.001, **** p < 0.0001; one-way ANOVA with Tukey post hoc test. (C) The expression of *PC* and *PCK2* throughout hepatic differentiation process. Data were normalized to the housekeeping gene ACTB and expressed relative to day 0 cells. The results shown represent three biological replicates and error bars represent SD. **p < 0.01, *** p < 0.001, **** p < 0.0001; two-way ANOVA with Tukey post hoc test.

gRNA name	Sequence (5’-3’)
HNF4α-KO-g1-F	caccGCTAGAGAAAGCTGGGGCTCG
HNF4α-KO-g1-R	aaacCGAGCCCCAGCTTTCTCTAGC
HNF4α-KO-g2-F	caccGAACCTGGCCACCTTCTGGGG
HNF4α-KO-g2-R	aaacCCCCAGAAGGTGGCCAGGTTC
HNF4α-KO-g3-F	caccGCCCAGAGACAGCTGCCCTTC
HNF4α-KO-g3-R	aaacGAAGGGCAGCTGTCTCTGGGC
HNF4α-KO-g4-F	caccGTCAGGGAGAAGACAGACCTT
HNF4α-KO-g4-R	aaacAAGGTCTGTCTTCTCCCTGAC
HNF4α-Kin-g11-F	caccGTGCAACAGGTTGTCAATCT
HNF4α-Kin-g11-R	aaacAGATTGACAACCTGTTGCAC
HNF4α-Kin-g12-F	caccGGGAGGTCCGTGCCAAGCCC
HNF4α-Kin-g12-R	aaacGGGCTTGGCACGGACCTCCC
**Name**	**Sequence (5’-3’)**
5-HA-F	gagctcgtttaaacggcgcgccGCTTGAACCCAGGAGGCA
5-HA-R	gcaatatttcaagaatgcaTAAACTGAATCTGCTCGATCATCTG
3-HA-F	gattatctttctagggttaacGCTCTTCGGCATGGCTAG
3-HA-R	cggcccggccttaattaaTCTCAGGAATCTGCAATTCAC

Table S5. Related to Figure 1, gRNA names and sequences (top panel) and primer names and sequences used in Gibson assembly.

Name	Sequence (5’-3’)
T7E1-KO-F	CCTGGTCCCTTGGCAAGAACT
T7E1-KO-R	AAGACGGGCTCATCTACTGAGGC
T7E1-Kin-F	TTGCCCACCCTCTTCCATTGT
T7E1-Kin-R	CACTTCAGAGAGGTCTCCTGTGGC
**Name**	**Sequence (5’-3’)**
targeting-LA-F1	CTATGCATGGTGCTAGGTGG
targeting-LA-R1	CATTTTGACTCACGCGGTCG
targeting-LA-R2	TCTCAGGAATCTGCAATTCACTC
targeting-RA-F1	CAGGTGCAGGTGAGCTTGGAGG
targeting-RA-F2	CAGACCGATAAAACACATGCG
targeting-RA-R1	GGATCTCTGAGGTCACCCCTCACTAC

Table S6. Related to Figure 1, Primer names and sequences (top panel) used in T7EI assay and primer names and sequences used in screening of knock-in clones.

Immunostaining Antibody Name	Host	Dilution	Supplier
**Primary antibodies**			

OCT4	Rabbit Poly	1/200	Abcam
NANOG	Goat Poly	1/200	Abcam
AFP	Mouse Mono	1/500	Abcam
SOX17	Goat Poly	1/500	R & D Systems
HNF4α	Rabbit Poly	1/100	Santa Cruz
IgG	Mouse	1/400	DAKO
IgG	Rabbit	1/400	DAKO
IgG	Goat	1/400	DAKO
**Secondary antibodies**			
Anti-Mouse 488	Rabbit	1/400	Life Technologies
Anti-Mouse 568	Goat	1/400	Life Technologies
Anti-Rabbit 488	Donkey	1/400	Life Technologies
Anti-Rabbit 568	Donkey	1/400	Life Technologies
Anti-Goat 488	Rabbit	1/400	Life Technologies
Anti-Goat 568	Rabbit	1/400	Life Technologies

**Flow Antibody**	**Conjugated**	**Type**	**Dilution**	**Supplier**
SSEA-1	FITC	Mouse IgM	1/50	Biolegend
SSEA-4	PE	Mouse IgG	1/50	Biolegend
TRA-1-60	PE	Mouse IgM	1/50	Biolegend

**Western Blot Antibody**	**Host**	**Dilution**	**Supplier**
**Primary antibodies**			
HNF4α	Rabbit Ploy	1/1000	Santa Cruz
b-actin	Mouse Mono	1/10000	Sigma
**Secondary antibodies**			
Anti-Rabbit IgG HRP	Swine	1/3000	R & D Systems
Anti-Mouse IgG HRP	Goat	1/3000	R & D Systems

Table S7. Related to Figure 1 and 2, Optimised antibody concentrations used in immunostaining, flow cytometry and western blotting.

Gene name	Probe number
HNF4α	Hs01023298_m1
HNF1α	Hs00167041_m1
TTR	Hs00174914_m1
APOA2	Hs00155788_m1
APOA4	Hs00166636_m1
HMGCS1	Hs00266810_m1
HMGCS2	Hs00985427_m1
LAMB3	Hs00165078_m1
FGA	Hs00241027_m1
FOXA2	Hs00232764_m1
SOX17	Hs00751752_s1
AFP	Hs01040607_m1
CEBPA	Hs00269972_s1
GLDC	Hs01580591_m1
GLUD1	Hs03989560_s1
ALDH5A1	Hs00542449_m1
PC	Hs00559398_m1
PCK2	Hs00356436_m1
ACTB	Hs01060665_g1

Table S8. Related to Figure 2 and 4, TaqMan probes used in qPCR.

TRANSPARENT METHODS

Detailed methods are provided in the online version of this paper and include the following:

- **KEY RESOURCES TABLE**
- **CONTACT FOR REAGENTS AND SOURCE SHARING**
- **EXPERIMENTAL MODEL AND SUBJECT DETAILS**
    - Cell lines
    - Cell Culture Conditions
    - Hepatocyte Differentiation System

- **METHOD DETAILS**
    - CRISPR/Cas9n-sgRNA expression plasmids and targeting vector construction
    - Nucleofection of H9 cells
    - Selection of the nucleofected cells
    - Genotyping the single cell-derived colonies
    - Removal of the transposon in the HNF4α point mutation knock-in experiment
    - Immunostaining
    - Flow cytometry
    - Western blotting
    - ELISA quantification
    - CYP3A assay
    - ^{1}H NMR-based metabolomics analysis
    - Microarray analysis
    - Label-free LC-MS/MS proteomics analysis
- **QUANTIFICATION AND STATISTICAL ANALYSIS**
    - Metabolomics data analysis
    - Microarray data processing and visualization
    - Gene set enrichment analysis (Gene Ontology)
    - Label-free proteomics data analysis
    - Visualization of over-layered analysis of microarray and proteomics datasets
- **DATA AND SOFTWARE AVAILABILITY**

KEY RESOURCES TABLE

**REAGENTS or RESOURCES**	**SOURCE**	**IDENTIFIER**
**Antibodies**		
Human NANOG antibody	R&D systems	AF1997
Human OCT4 antibody	Abcam	AB19857
Human SOX17 antibody	R&D systems	AF1924
Anti-Human SSEA4 PE	eBioscience	12-8843-42

Anti-Human CD15(SSEA1) FITC	eBioscience	11-0159-42
Anti-Human TRA-1-60 PE	eBioscience	12-8863-82
HNF4α (H-171)	SantaCruz	Sc-8987
Monoclonal anti-AFP antibody	Sigma	A8452
Monoclonal anti-β-ACTIN	R&D systems	A5441
Mouse IgM isotype control FITC	eBioscience	11-4752-80
Mouse IgG3 isotype control PE	eBioscience	12-4742-41
Polyclonal Swine Anti-Rabbit Immunoglobulins HRP	DAKO	P0399
Polyclonal Goat Anti-Mouse Immunoglobulins HRP	DAKO	P0447
Donkey anti-Goat IgG (H+L) Cross-Adsorbed, Alexa Fluor® 568	Thermo Fisher Scientific	A11057
Goat anti-Rabbit IgG (H+L) Cross-Adsorbed, Alexa Fluor® 488	Thermo Fisher Scientific	A11008
Donkey anti-Mouse IgG (H+L) Highly Cross-Adsorbed, Alexa Fluor® 488	Thermo Fisher Scientific	A21202
4’,6-Diamidine-2’-phenylindole dihydrochloride (DAPI)	Roche	10236276001
Mouse IgG	Vector Laboratories	I-2000
Rabbit IgG	Vector Laboratories	I-1000

Goat IgG	Vector Laboratories	I-5000
**Bacterial and Virus Strains**		
One Shot TOP10 Chemically Competent *E. coli*	Thermo Fisher Scientific	C404003
One Shot Stbl3 chemically competent *E.coli*	Thermo Fisher Scientific	C7373-03
**Plasmid Constructs**		
pSpCas9n(BB)-2A-Puro (PX462)	Addgene	PX462
pMCS-AAT_PB-PGKpuroTK	Wellcome Trust Sanger Institute	N/A
pCMV-hyPBase	Wellcome Trust Sanger Institute	N/A
pMD19-T vector	Takara	3271
**Chemicals, Peptides, and Recombinant Proteins**		
Matrigel matrix	Corning	354248
Human Recombinant Laminin 521	BioLamina	LN521
Human Recombinant Laminin 111	BioLamina	LN111
Recombinant Human/Murine/Rat Activin A (*E.coli* derived)	Peprotech	120-14E
Recombinant Mouse Wnt-3a Protein	R&D systems	1324-WN/CF
Recombinant Human HGF	Peprotech	100-39
Recombinant Human Oncostatin M	Peprotech	300-10

B27 minus vitamin A	Thermo Fisher Scientific	12587-010
RPMI 1640	Thermo Fisher Scientific	11875-093
Penicillin-Streptomycin	Thermo Fisher Scientific	15140-122
Knockout DMEM	Thermo Fisher Scientific	10829-018
Knockout serum replacement	Thermo Fisher Scientific	10828-028
GlutaMAX	Thermo Fisher Scientific	35050-038
NEAA	Thermo Fisher Scientific	11140-035
DMSO	Honeywell Chemicals	D5879
Beta-mecaptoethanol	Thermo Fisher Scientific	31350-010
HepatoZYME-SFM	Thermo Fisher Scientific	17705-021
Hydrocortisone-21 hemisuccinate sodium salt	Sigma	H4881
mTeSR1 medium	Stem Cell Technologies	85850
Gentle Cell Dissociation Reagent	Stem Cell Technologies	07174
TrypLE Express Enzyme	Thermo Fisher Scientific	12604013
Y-27632 (Dihydrochloride)	Millipore	SCM075
DPBS	Thermo Fisher Scientific	14190144

DPBS with calcium and magnesium	Thermo Fisher Scientific	14040133
Bovine Serum Albumin	Sigma	A7906-10G
OneComp eBeads 100 tests	eBioscience	01-1111-42
Trypan Blue Solution, 0.4%	Thermo Fisher Scientific	15250061
NuPAGE 4-12% Bis-Tris Protein Gels, 1.5mm, 10-well	Thermo Fisher Scientific	NP0335BOX
NuPAGE MES SDS Running Buffer (20x)	Life Technologies	NP0002
NuPAGE Transfer Buffer (20x)	Life Technologies	NP0006-1
NuPAGE LDS Sample Buffer	Thermo Fisher Scientific	NP0007
SeeBlue Plus 2 Prestained Standard	Thermo Fisher Scientific	LC5925
TWEEN 20	Sigma	T2700-100ML
Phosphate buffered saline tablet	Sigma	P4417-100TAB
SYBR Safe DNA gel stain	Life Technologies	S33102
UltraPure Agarose	Life Technologies	16500-100
DNA ladder, 1 kb	New England BioLabs	N3232S
UltraPure DNase/RNase-free distilled water	Life Technologies	10977-023
Ampicillin	Sigma	A9518-5G
SOC medium	New England BioLabs	B9020S
LB medium	Sigma	L3022
LB agar medium	Sigma	L2897
FIAU	Moravek	M251

Puromycin dihydrochloride	Life Technologies	A11138-03
FastDigest BbsI (BpiI)	Fermentas/Thermo Scientific	FD1014
DTT	Fermentas/Thermo Scientific	R0862
T4 DNA ligase and buffer	New England BioLabs	M0202S
T4 polynucleotide kinase	New England BioLabs	M0201S
Adenosine 5′-triphosphate, 10 mM	New England BioLabs	P0756S
Q5 High-Fidelity DNA Polymerases	New England BioLabs	M0491
T7 endonuclease I (T7EI)	New England BioLabs	M0302S
NsiI-HF	New England BioLabs	R3127S
AscI	New England BioLabs	R0558S
HpaI	New England BioLabs	R0105S
PacI	New England BioLabs	R0547S
TRIzol Reagent	Thermo Fisher Scientific	15596026
**Critical Commercial Assays**		
Human stem cell Nucleofector kit 1	Lonza	VPH-5012
P450-Glo™ CYP3A4 Assay System	Promega	V8902
Human Alpha Fetoprotein (AFP) ELISA Kit, 96 tests	Alpha Diagnostic	0500

Human Serum Albumin ELISA Kit, 96 tests	Alpha Diagnostic	1190
GenElute Mammalian Genomic DNA miniprep Kit	Sigma	G1N70-1KT
Pierce BCA Protein Assay Kit	Thermo Fisher Scientific	23225
SuperSignal West Pico Chemiluminescent Substrate	Thermo Fisher Scientific	34077
QIAprep spin miniprep kit	Qiagen	27106
QIAquick gel extraction kit	Qiagen	28704
GeneChip Human Genome HG-U133 Plus 2.0 Array	Affymetrix	N/A
**Experimental Models: Cell Lines**		
H9 female human ESCs	WiCell	WA09
H9 with HNF4α truncated	This study	N/A
H9 with HNF4α point mutated	This study	N/A
**Oligonucleotides**		
See Table S5, S6	N/A	N/A
**Recombinant DNA**		
gRNA for CRISPR cloned in SpCas9n(px462), see Table S5 for oligonucleotide sequences	This study	N/A
PB_HNF4α_PGK puroTK	This study	N/A
**Software and Algorithms**		

Flowjo	FLOWJO, LLC	https://www.flowjo.com/
FACSDIVA software	BD Biosciences	N/A
GraphPad Prism	GraphPad Software	https://www.graphpad.com/scientific software/prism/
Columbus Image Data Storage and Analysis System	PerkinElmer	http://www.cambridgesoft.com/ensemble/spotfire/Columbus/default.aspx
Chenomx NMR Suite version 8.1	Chenomx	https://www.chenomx.com/software/archive/
Microarray Suite software	Affymetrix	N/A
Limma packages	Bioconductor	https://bioconductor.org/packages/ release/bioc/html/limma.html
MaxQuant proteomics software	Max Planck Institute of Biochemistry	http://www.biochem.mpg.de/5111795/maxquant
Perseus proteomics software	Max Planck Institute of Biochemistry	http://www.biochem.mpg.de/5111810/perseus
Enrichr analysis website	Icahn School of Medicine at Mount Sinai	http://amp.pharm.mssm.edu/Enrichr/
STRING protein interaction analysis website	N/A	https://string-db.org
PaintOmics3	N/A	http://bioinfo.cipf.es/paintomics/

CONTACT FOR REAGENTS AND SOURCE SHARING

Further information and requests for resources and reagents should be directed to and will be fulfilled by the Lead Contact, David C. Hay (davehay@talktalk.net).

EXPERIMENTAL MODEL AND SUBJECT DETAILS

Cell lines

H9 female human ESCs were purchased from WiCell. Cells were routinely checked for cell surface markers using flow cytometry and for the presence of mycoplasma.

Cell Culture Conditions

H9 human ESCs and genome modified DBD Mut and SUMO Mut cell lines were cultured on Matrigel coated cell culture plates with mTeSR1 medium. Cells were routinely split in 1:3 ratio

using Gentle Cell Dissociation reagent. For nucleofection, single cells were obtained by treating the cells with Gentle Cell Dissociation reagent for 7-8 minutes at 37 ^{o}C. ROCK inhibitor was used to maintain the single cells.

Hepatocyte Differentiation System

Hepatocyte differentiation of the different cell lines was carried out following lab-developed protocol (Wang et al., 2017). Briefly, 5.5-6.5x10^{5} single cells were seeded into per well on 6-well plates coated with LN521/LN111 mix (1:3) for differentiation. Once cells reached approximately 40% confluency post seeding, hepatocyte differentiation was initiated by switching the mTeSR1 medium to RPMI 1640 medium supplemented with 2 % B27 (minus Vitamin A), 100 ng/ml Activin A and 50 ng/ml Wnt 3a. This definitive endoderm induction medium was changed daily for 3 days. Subsequently, the differentiation medium was switched to Knockout DMEM medium supplemented with 20 % knockout serum replacement, 0.5 % GlutaMAX, 1% NEAA and DMSO, and 0.1 mM beta mecaptoethanol. This hepatic progenitor specification medium was changed daily for 5 days. Following this, the differentiation medium was changed to HepatoZYME medium supplemented with 1 % GlutaMAX, 10 uM hydrocortisone, 20 ng/ml hepatocyte growth factor (HGF) and 10 ng/ml oncostatin M (OSM). This hepatocyte-like cells maturation medium was changed every other day for 10 days. The derived hepatocyte-like cells were checked for functions, including basal cytochrome P450 3A activity, albumin and AFP secretion. In addition, cells at representative time points (day 0, day 3, day 9 and day 18) were routinely checked for the expression of typical markers corresponding to that time point.

METHOD DETAILS

CRISPR/Cas9n-sgRNA expression plasmids and targeting vector construction

Oligonucleotides for gRNAs (Table S5) targeting HNF4α were annealed and cloned into a BbsI digested Cas9n backbone plasmid (PX462) following a published protocol (Ran et al., 2013).

The construction of the *piggyBac*-based targeting construct involved three main steps: the amplification of the 5'-homology arm (HA) and the 3'-HA; the insertion of the 5'-HA into the backbone plasmid; and the insertion of the 3'-HA into the backbone plasmid.

The PCR primers for amplifying 5'-HA and 3'-HA used in Gibson assembly were generated using NEBuilder (https://nebuilder.neb.com) (Table S5). The desired point mutations were incorporated into the primers. The PCR amplification of the two homology arms were catalysed by Q5 high-fidelity DNA polymerase (New England Biolabs). H9 genomic DNA was used as the template for the PCR. The amplified homology arms were then purified before being inserted into the backbone plasmid.

The used *piggyBac* backbone was a kind gift of Dr. Kosuke Yusa from the Wellcome Trust Sanger Institute, UK. For the insertion of the 5'-HA, the backbone plasmid was digested using NsiI and AscI enzymes and then gel purified. The purified 5'-HA was then inserted using a Gibson assembly reaction. The *piggyBac*-5'-HA plasmid was then purified and sequenced to confirm the correct insertion of the 5'-HA. Following this, the *piggyBac*-5'-HA plasmid was digested using HpaI and PacI enzymes and then purified, the 3'-HA was then inserted into the

backbone and completed the construction of the donor plasmid. Sequencing was also performed to confirm the correct insertion of the 3'-HA.

Nucleofection of H9 cells

H9 cells at 70-80 % confluency were dissociated using Gentle Cell Dissociation reagent to make single cell suspension. For HNF4α gene knockout, ~ 0.8 million live cells were nucleofected with 3 μg of each Cas9n-sgRNA expression plasmid. For the knockin experiment, ~ 0.8 million cells were nucleofected with 5 μg donor plasmid and 3 μg of both Cas9n-sgRNA expression plasmids. The Human Stem Cell Nucleofector® Kit 1 (Lonza) was used and the nucleofection was facilitated by a Nucleofector 2b device with program A-023 (Lonza).

Selection of the nucleofected cells

48 hours post nucleofection, the cells were selected using 0.5 μg/ml puromycin for 2 days. Puromycin-resistant cells were then propagated and passaged to 10-15 96-well plates at the concentration of 0.8 cell/well in mTeSR1 medium supplemented with 10 μM Rho-associated protein kinase (ROCK) inhibitor (Sigma-Aldrich). At this point, puromycin in the medium for HNF4α knockout experiment cells was removed, but it was kept for selecting cells for HNF4α point mutation knock-in experiment. Following the seeding, the cells were maintained at 37 ^{o}C with 10 % CO2 for 7 days to form single cell-derived colonies.

The single-cell derived colonies were picked at about another 5 days later and passaged at a 1:2 ratio to two different plates. ~4 days after the passaging, cells on one plate were lysed for genomic DNA extraction.

Genotyping the single cell-derived colonies

Colonies from HNF4α knockout experiments were analysed using T7EI assay (Table S6). The targeted region was PCR amplified and the PCR products were then digested using T7EI enzyme, and subsequently separated on agarose gels and the genotypes were 'readable' on the gel.

Colonies from HNF4α point mutation knock-in experiments were amplified using three-primer PCRs (Table S6) to differentiate genomic sequences and inserted sequences. The PCR products were separated on agarose gels and the genotype were 'readable' on the gel.

For promising colonies from the DNA gel-based genotyping, the PCR products were purified and then inserted into a pMD19-T vector to form a stable plasmid for sequencing. The colonies with correct genotypes were then saved and expanded to form stable isogenic cell lines.

Removal of the transposon in the HNF4α point mutation knock-in experiment

The correctly targeted colonies were established and two were used for the removal of the selection cassette introduced by the transposon. Briefly, ~0.8 million cells were nucleofected with 5 μg hyperactive PBase (gift from Dr. Kosuke Yusa) and the cells were culture in mTeSR1 medium without puromycin. The cells were then maintained for 3-4 passages before the negative selection mediated by the thymidine analog 1-(2-deoxy-2-fluoro-β-D-arabinofuranosyl)-5-iodouracil (FIAU) at a concentration of 200 nM. About 2 weeks after the

negative selection, single cell-derived colonies were picked and genotyped as described above.

Immunostaining

Cells were fixed in 100 % ice-cold methanol at -20 °C for 30 minutes. Post fixation, cells were washed three times with DPBS for 5 minutes at room temperature. The cells were subsequently blocked with PBS-0.1 % Tween (PBST) containing 10 % BSA for 1 hour at room temperature. After that, the blocking solution was removed and the respective primary antibody diluted in 1% BSA/PBST was added to the cells. The primary antibody was incubated at 4 °C overnight with gentle agitation. Following this, the used primary antibody solution was removed and the cells were washed three times with PBST for 5 minutes at room temperature. The cells were then incubated with the appropriate secondary antibody diluted in PBST for 1 hours at room temperature in the dark with gentle agitation. The cells were then washed three times with PBST. Following this, cells were stained with DAPI diluted in PBS (1:5000) for 2 minutes at room temperature. The DAPI solution was then removed and the cells were washed twice with PBST. In all cases, the stained cells were stored at 4 °C in the dark before imaging. The primary and secondary antibodies are listed in supplemental Table S7.

All images were collected at room temperature using the automated Operetta fluorescent microscope. The images were processed using Columbus Image analysis server.

Flow cytometry

Fluorescence activated cell sorting (FACS) was used to confirm the expression of cell surface markers in hESCs. hESCs from a well on a 6-well plate were washed once with 2 ml DPBS and then dissociated with 1ml TrypLE. The cells were lifted off as single cells and collected. Post centrifugation, cells were resuspended in DPBS and filtered through a 0.22 μm filter. The single cell suspension was then incubated with the fluorochrome conjugated antibodies and the corresponding IgG controls for 30 minutes at 4 °C. The antibody information is listed in supplemental Table S7. Cells were then washed twice with DPBS, removing any unbound antibody, and span down at 200 x g for 5 minutes. Following this, cells were resuspended in 300 μl of DPBS.

Dead cells and debris were not included in the analysis. This was carried out by using an electronic live gate on forward scatter and side scatter parameters. Data for 20,000-50,000 'live' events were acquired for each sample using a BD LSR Fortessa (4 laser) analyser. The data was analysed using FlowJo software.

Western blotting

Cells at different time points were washed once with DPBS and lysed in wells at room temperature in dark for 5 min. 200 μl home-made SUMO lysis buffer (2 % SDS, 50 mM Tris-HCL (pH 8.0), 1 mM EDTA, 10 mM iodoacetamide) supplemented with proteinase and phosphatase inhibitors (Sigma-Aldrich) at 1 % final concentration was used for each well of a 6-well plate. The cell extract was briefly sonicated using a Bioruptor (Diagenode) and span down for 15 min at 12,000 rpm at 4 °C. The protein supernatant was then transferred to a new 1.5 ml Eppendorf tube.

The Pierce bicinchoninic acid (BCA) assay kit (Thermo Fisher Scientific) was used to quantify the protein concentration. Protein supernatant was diluted 1:10 using the lysis buffer. 20 μl protein supernatant of each sample was analysed in duplicates. Reagent A and B from the kit were mixed at a 1:50 ratio and a volume of 200 μl was transferred into each sample well. A standard curve was generated using the bovine serum albumin standards at concentrations ranging from 0-2000 μg/ml. The plate was incubated at room temperature for 30 min and the absorbance was read at 562 nm. The protein concentrations were calculated by linear extrapolation using the standard curve generated.

The SDS-NuPAGE polyacrylamide gel electrophoresis (SDS-PAGE) was used to separate proteins of different sizes. 50 μg protein supernatant was denatured at 100 ^{o}C for 10min in 1x NuPAGE LDS sample buffer supplemented with 10 mM DTT before being used for electrophoresis. 4-12 % Bis-Tris precast polyacrylamide gels (Thermo Fisher Scientific) were used with the XCell SureLock Mini-Cell System (Thermo Fisher Scientific) for the electrophoresis. Once the gel was fitted in the chamber, the tank was filled with 1x NuPAGE MES-SDS running buffer with 0.5 ml of NuPAGE Antioxidant (Thermo Fisher Scientific) added into the inner chamber. The samples were loaded along with a SeeBlue Plus 2 Pre-Stained Standard (Invitrogen). A current of 200 V was applied and the samples were run for 1-2 hours depending on the purpose of each experiment. The gels containing proteins were carefully removed from the cassette and were used in western blotting.

Following the protein separation using SDS-PAGE gels, the proteins were then transferred from the gels to the Polyvinylidene fluoride (PVDF) membrane. The transfer sack was assembled in the following order from cathode to anode: 3x sponge; 2x filter paper soaked in 1x NuPAGE Transfer Buffer; SDS-PAGE gel; PVDF membrane activated in 100 % ice-cold methanol; 2x filter paper soaked in 1x NuPAGE Transfer Buffer; 3x sponge. No bubbles between the gel and the membrane were allowed. Once the sack was assembled and placed into the XCell Blot II module, the module was then tightly sealed and placed into the transfer SureLock tank containing 1x transfer buffer in the inner chamber and ice-cold water in the outer chamber. The tank was then placed on ice for the entire protein transfer process. A constant current of 160mA was applied for 90-120 min.

Once the proteins were successfully transfer onto the PVDF membrane, the membrane was blocked in 10 % skimmed milk at room temperature with gentle agitation for at least one hour to prevent non-specific antibody binding.

Following this, the membrane was probed with desired primary antibody diluted in 5 ml 10 % BSA/PBST at 4 ^{o}C overnight with gentle rolling. The dilution ratios were optimised specifically for each primary antibody and were listed in Table 4. Unbound antibody was removed by three, 5-min washes with adequate 0.1 % PBST at room temperature with agitation. The primary antibody could be re-used for 2-3 times if stored at 4 ^{o}C properly.

After probed with the primary antibody, the membrane was then incubated with corresponding horseradish peroxidase (HRP)-conjugated secondary antibody diluted in 5 ml 10 % skimmed milk at room temperature for at least one hour with gentle rolling. Unbound secondary antibody was then removed by three, 5-min washes with adequate 0.1 % PBST at

room temperature with agitation. The primary and secondary antibody information is listed in supplemental Table S7.

The proteins of interest were then detected using enhanced chemiluminescence (ECL). Proteins bands were visualised using the Pierce Enhanced Chemiluminescence Kit (Pierce, UK). Peroxidase buffer and the Luminol/Enhancer solution was mixed at a 1:1 ratio and spread evenly onto the membrane (2 ml for each membrane), followed by a 5-min incubation at room temperature. The HRP substrate reacts with the conjugated HRP group presented on the secondary antibody, specifying the target protein. The membrane was developed in the dark room using a film developer.

ELISA quantification
At the hepatocyte stage, day 18 cell culture supernatants were collected. Albumin and AFP concentration were determined using commercially available kits (Alpha Diagnostic Intl. Inc, San Antonio, USA). The ELISA assay was performed according to the manufacturer's instructions with cell culture supernatants diluted at 1:3 (albumin) or 1:10 (AFP). After one hour incubation at room temperature, the wells were washed for four times and anti-human albumin or AFP HRP-conjugated secondary antibodies were added to the wells for 30 minutes. Subsequently, the wells were washed five times and the substrate was then added and incubated for a further 15 minutes in the dark at room temperature. Following this, the stop solution was added directly to the wells in order to stop the enzymatic reaction. The plates were then read at 450 nm with a reference wavelength of 630nm using a FLUOstart Omega plate reader (BMG LabTech, Germany). Blank cell culture media incubated at 37 ^{o}C for 48 hours, diluted in 1:3 or 1:10 was used as a negative control. The data was then normalised to per ml per 48 hours per mg protein as determined by BCA assay (Pierce, UK).

Quantitative real-time PCR
Total RNA was collected from cells using TRIzol reagent (Invitrogen) according to the manufacturer's instructions. Total RNA was reverse transcribed using QuantiTect reverse-transcription kit (Qiagen) following the manufacturer's protocol. Real-time PCR was performed using TaqMan Fast Universal Master Mix and appropriate Taqman probes (Applied Biosystems). The primers are listed in supplemental Table S8. The samples were analysed using Roche LightCycler 480 Real-Time PCR System. Results were normalized to beta-actin. The qPCRs were run in triplicates.

CYP3A assay
Day 18 hESC-derived hepatocyte-like cells (HLCs) were incubated with the luciferin conjugated CYP3A (1:40) substrate (P450 P-Glo Luminescent Kit, Promega, UK) diluted in the maturation medium for 5 hours at 37 ^{o}C. Blank cell culture medium was used as a negative control. The supernatants were then collected and tested for luminescence. The luciferin detection reagent was reconstituted by mixing the buffer with the lyophilised luciferin detection reagent. 50 μl of the collected supernatant was mixed with 50 μl of the detection reagent in a white 96 well plate and incubated at room temperature in the dark for 20 minutes. The relative levels of basal activity were measured using a luminometer (POLARstar optima). Units of activity were recorded as relative light units per ml per mg protein (RLU/ml/mg) as normalised by protein content.

^{1}H NMR-based metabolomics analysis

24-hour cell culture medium from cells at differentiation day 9 was collected. The medium was then centrifuged at 4 °C, 300 x g for 5 min. The supernatant was collected and flash-frozen on dry ice.

All media samples were analysed on a 700 MHz Bruker Avance Neo NMR spectrometer equipped with a BBI room temperature probe. The aliquots of the conditioned media from each cell line and unconditioned controls were thawed at room temperature and 550 μL of each was transferred into a microcentrifuge tube, to this 50 μL of deuterium oxide containing 1 mM deuterated 3-(Trimethylsilyl)-1-propanesulfonic acid-d6 sodium (DSS-d6, 98% atom %D, Sigma-Aldrich), which was used as an internal reference for calibration, was added. The total volume of 600 μL was transferred into individually coded sample tubes in 96-racks (Bruker).

Briefly, the samples were loaded using a SampleJet sample changer. The samples remained chilled at 277 K on the sample changer and at 300 K during acquisition of the ^{1}H NMR experiments. Matching, tuning and shimming were performed automatically on each individual sample to reduce sample-to-sample variation. ^{1}H NMR spectra were collected using a standard 1D-nuclear overhauser enhancement spectroscopy (NOESY)-presaturation and THE PROJECT-Carr-Purcell-Meiboom-Gill (CPMG) pulse sequences. The RF field strength for presaturation was 25Hz. For both experiments 8 scans were collected with a spectral width of 20 ppm, an acquisition time of 3.0 s and a relaxation delay of 40.0 s, which was determined from T1 measurements. The value of O1 was determined automatically for the first 1D-NOESY experiment and was used subsequently for the CPMG experiment. The total CPMG period was 64 ms with 180^0 every 400 μs. For both experiments a signal-to-noise ratio of greater than 100:1 was achieved. A range of metabolites were identified with the aid of the software Chenomx NMR Suite version 8.1 (Chenomx Inc, Edmonton, Alberta) and human metabolome database (HMDB).

Microarray analysis

Cells at differentiation day 9 on Laminin 521/111 mix (1:3 ratio) were lysed in TRIZOL and stored at -80 °C. RNA extraction was performed according to the manufacturer's instructions. Analysis of gene expression was performed using Affymetrix GenChip Human Genome HG-U133 plus 2.0 array (Santa Clara, CA, USA). Microarrays were scanned with an Affymetrix scanner controlled by Affymetrix Microarray Suite software.

Label-free LC-MS/MS proteomics analysis

To facilitate whole proteome analysis of the three lines, quadruplicate samples of WT, DBD Mut and SUMO Mut cells were taken at differentiation day 9. Total protein extract was prepared in 1x LDS sample buffer, and 20 μg total protein for each sample was sonicated and boiled for 10 minutes prior to fractionation by NuPAGE 10% Bis-Tris gels using 3-(N-morpholino) propanesulfonic acid (MOPS) buffer. Each lane of the gel was excised into 4 slices, and peptides were prepared in parallel by in-gel tryptic digestion (Shevchenko et al., 2007). For each sample, approximately 1μg total peptide was submitted for liquid chromatography coupled tandem MS (LC-MS/MS) analysis on a Q Exactive mass spectrometer (Thermo Scientific) coupled to an EASY-nLC 1000 liquid chromatography system (Thermo Scientific). Peptides were fractionated on a 75 μm x 500 mm EASY-Spray column (Thermo

Scientific) using a 90-minute gradient. Precursor ion full scan spectra were acquired over (m/z 300 to 1,800) with a resolution of 70,000 at m/z 200 (target value of 1,000,000 ions, maximum injection time 20 ms). Up to ten data dependent MS2 spectra were acquired with a resolution of 35,000 at m/z 200 (target value of 1,000,000 ions, maximum injection time 120 ms). Ions with unassigned, +1 and +8 charge state were rejected. Intensity threshold was set to 2.1 x 10^4 units. Peptide match was set to preferred, dynamic exclusion duration 40 s.

QUANTIFICATION AND STATISTICAL ANALYSIS

Metabolomics data analysis

The metabolic rate for each metabolite was quantified by normalizing the signal intensity against the cell number at the detected time point. The unit for the metabolic rate is then recorded as rate of integral area per cell. A negative rate indicates consumption, while a positive value means production of the metabolite. All samples were tested in triplicates.

Microarray data processing and visualization

Affymetrix gene expression data were pre-processed using 'affyPLM' packages of the Bioconductor Software (Gentleman et al., 2004). To obtain the genes with the strongest evidence of differential expression, a linear model fit was applied for each gene using 'limma' (Linear Models for Microarray Data) packages of the Bioconductor Software.

Gene expression levels in each HNF4α-edited cell line were log2 transformed and then compared to the wild type control cells. Genes with a fold change greater than two over the wild type cell expression levels (p value <= 0.05, false discovery rate (1% FDR) corrected) were taken as significantly deregulated (Table S1). All samples were tested in triplicates. Processing and visualization (Principal Component Analysis) of data were performed using MATLAB tools (The MathWorks Inc., Natick, MA, USA).

Gene set enrichment analysis (Gene Ontology)

Genes which showed change of equal to or more than 2-fold were subjected to gene ontology (GO) analyses using the web server Enrichr (Chen et al., 2013; Kuleshov et al., 2016). The results of biological pathways (GOBP) were provided in Table S2.

Label-free proteomics data analysis

Data were analyzed using MaxQuant (version 1.5.8.3) (Cox and Mann, 2008; Cox et al., 2011) and searched against UniProtKB *H. sapiens* database (86749 sequences - 13/06/2012). Unless otherwise stated, all MaxQuant settings were default. Variable modifications of acetyl (Protein N-term) and oxidation (M) were considered, along with the fixed modification of carbamidomethyl (C). Enzyme specificity was set to trypsin/P, and a false discovery rate of 1 % was set as a threshold at protein, peptide and site levels, and a mass deviation of 6 ppm was set for main search and 20 ppm for MS2 peaks. Match between runs was applied, and label-free quantification (LFQ) was selected.

The MaxQuant proteinGroups.txt file was used for downstream proteome analysis. All decoy and 'putative contaminant' entries were removed, as were any identified only by site and those with fewer than 4 reported LFQ values for any single experimental group (WT, DBD Mut

and SUMO Mut). This left 3639 protein groups comparable across all cell types. Zero LFQ values were replaced in Perseus (Tyanova et al., 2016), from a normal distribution of existing $\log_{10}$ LFQ intensities downshifted 1.8 and of width 0.3. Proteins were defined as statistically differing between groups using the Perseus unpaired two-samples Student's t-test truncated by 1% permutation-based FDR using an S0 value of 0.1. No apparent specificity was entered as an empty cell in the spreadsheet.

PCA plotting was performed using Perseus software. GOBP enrichment analysis was performed on Enrichr server (Table S4).

Protein-protein interaction visualization was performed using a web server named STRING (https://string-db.org).

Visualization of over-layered analysis of microarray and proteomics datasets
A free web-based multi-omics data visualization application named PaintOmics 3 (http://bioinfo.cipf.es/paintomics/) (Hernandez-de-Diego et al., 2018) was used to do the over-layered analysis of microarray and proteomics datasets.

DATA AND SOFTWARE AVAILABILITY

Data availability

The accession number for the microarray data reported in this paper is EMBL-EBI: E-MTAB-7951.

The mass spectrometry proteomics data have been deposited to the ProteomeXchange Consortium via the PRIDE (Perez-Riverol et al., 2019) partner repository with the dataset identifier PXD013737.

REFERENCE

Chen, E.Y., Tan, C.M., Kou, Y., Duan, Q., Wang, Z., Meirelles, G. V., Clark, N.R., and Ma'ayan, A. (2013). Enrichr: Interactive and collaborative HTML5 gene list enrichment analysis tool. BMC Bioinformatics *14*.

Cox, J., and Mann, M. (2008). MaxQuant enables high peptide identification rates, individualized p.p.b.-range mass accuracies and proteome-wide protein quantification. Nat. Biotechnol. *26*, 1367–1372.

Cox, J., Neuhauser, N., Michalski, A., Scheltema, R.A., Olsen, J. V., and Mann, M. (2011). Andromeda: A peptide search engine integrated into the MaxQuant environment. J. Proteome Res. *10*, 1794–1805.

Gentleman, R., Carey, V., Bates, D., Bolstad, B., Dettling, M., Dudoit, S., Ellis, B., Gautier, L., Ge, Y., Gentry, J., et al. (2004). Bioconductor: open software development for computational biology and bioinformatics. Genome Biol. *5*, R80.

Hernandez-de-Diego, R., Tarazona, S., Martinez-Mira, C., Balzano-Nogueira, L., Furio-Tari, P., Pappas, G.J., and Conesa, A. (2018). PaintOmics 3: a web resource for the pathway analysis and visualization of multi-omics data. BioRxiv 1–7.

Kuleshov, M. V., Jones, M.R., Rouillard, A.D., Fernandez, N.F., Duan, Q., Wang, Z., Koplev, S., Jenkins, S.L., Jagodnik, K.M., Lachmann, A., et al. (2016). Enrichr: a comprehensive gene set enrichment analysis web server 2016 update. Nucleic Acids Res. *44*, W90–W97.

Perez-Riverol, Y., Csordas, A., Bai, J., Bernal-Llinares, M., Hewapathirana, S., Kundu, D.J., Inuganti, A., Griss, J., Mayer, G., Eisenacher, M., et al. (2019). The PRIDE database and related tools and resources in 2019: Improving support for quantification data. Nucleic Acids Res. *47*, D442–D450.

Ran, F.A., Hsu, P.D., Wright, J., Agarwala, V., Scott, D.A., and Zhang, F. (2013). Genome engineering using the CRISPR-Cas9 system. Nat. Protoc. *8*, 2281–2308.

Shevchenko, A., Tomas, H., Havliš, J., Olsen, J. V., and Mann, M. (2007). In-gel digestion for mass spectrometric characterization of proteins and proteomes. Nat. Protoc. *1*, 2856–2860.

Tyanova, S., Temu, T., Sinitcyn, P., Carlson, A., Hein, M.Y., Geiger, T., Mann, M., and Cox, J. (2016). The Perseus computational platform for comprehensive analysis of (prote)omics data. Nat. Methods *13*, 731–740.

Wang, Y., Alhaque, S., Cameron, K., Meseguer-Ripolles, J., Lucendo-Villarin, B., Rashidi, H., and Hay, D.C. (2017). Defined and Scalable Generation of Hepatocyte-like Cells from Human Pluripotent Stem Cells. J. Vis. Exp. 1–8.
